# Impact of hospital-specific domain adaptation on BERT-based models to classify neuroradiology reports

**DOI:** 10.1007/s00330-025-11500-9

**Published:** 2025-03-17

**Authors:** Siddharth Agarwal, David Wood, Benjamin A. K. Murray, Yiran Wei, Ayisha Al Busaidi, Sina Kafiabadi, Emily Guilhem, Jeremy Lynch, Matthew Townend, Asif Mazumder, Gareth J. Barker, James H. Cole, Peter Sasieni, Sebastien Ourselin, Marc Modat, Thomas C. Booth

**Affiliations:** 1 School of Biomedical Engineering & Imaging Sciences, King’s College London, Becket House, London, UK; 2 Department of Neuroradiology, Ruskin Wing, King’s College Hospital NHS Foundation Trust, London, UK; 3 Guy’s and St Thomas’ NHS Foundation Trust, Great Maze Pond, London, UK; 4 Department of Neuroimaging, Institute of Psychiatry, Psychology, & Neuroscience, King’s College London, London, UK; 5 Centre for Medical Image Computing, Department of Computer Science, University College London, London, UK; 6 Clinical Trials Unit, King’s College London, Guy’s Campus, Great Maze Pond, London, UK

**Keywords:** Neuroradiology, Natural language processing, Transformers, Language modelling, Pretraining

## Abstract

**Objectives:**

To determine the effectiveness of hospital-specific domain adaptation through masked language modelling (MLM) on BERT-based models’ performance in classifying neuroradiology reports, and to compare these models with open-source large language models (LLMs).

**Materials and methods:**

This retrospective study (2008–2019) utilised 126,556 and 86,032 MRI brain reports from two tertiary hospitals—King’s College Hospital (KCH) and Guys and St Thomas’ Trust (GSTT). Various BERT-based models, including RoBERTa, BioBERT and RadBERT, underwent MLM on unlabelled reports from these centres. The downstream tasks were binary abnormality classification and multi-label classification. Performances of models with and without hospital-specific domain adaptation were compared against each other and LLMs on internal (KCH) and external (GSTT) hold-out test sets. Model performances for binary classification were compared using 2-way and 1-way ANOVA.

**Results:**

All models that underwent hospital-specific domain adaptation performed better than their baseline counterparts (all *p*-values < 0.001). For binary classification, MLM on all available unlabelled reports (194,467 reports) yielded the highest balanced accuracies (KCH: mean 97.0 ± 0.4% (standard deviation), GSTT: 95.5 ± 1.0%), after which no differences between BERT-based models remained (1-way ANOVA, *p*-values > 0.05). There was a log-linear relationship between the number of reports and performance. LLama-3.0 70B was the best-performing LLM (KCH: 97.1%, GSTT: 94.0%). Multi-label classification demonstrated consistent performance improvements from MLM for all abnormality categories.

**Conclusion:**

Hospital-specific domain adaptation should be considered best practice when deploying BERT-based models in new clinical settings. When labelled data is scarce or unavailable, LLMs can serve as a viable alternative, assuming adequate computational power is accessible.

**Key Points:**

***Question*** *BERT-based models can classify radiology reports, but it is unclear if there is any incremental benefit from additional hospital-specific domain adaptation*.

***Findings*** *Hospital-specific domain adaptation resulted in the highest BERT-based model accuracies and performance scaled log-linearly with the number of reports*.

***Clinical relevance*** *BERT-based models after hospital-specific domain adaptation achieve the best classification results provided sufficient high-quality training labels. When labelled data is scarce, LLMs such as Llama-3.0 70B are a viable alternative provided there are sufficient computational resources*.

**Graphical Abstract:**

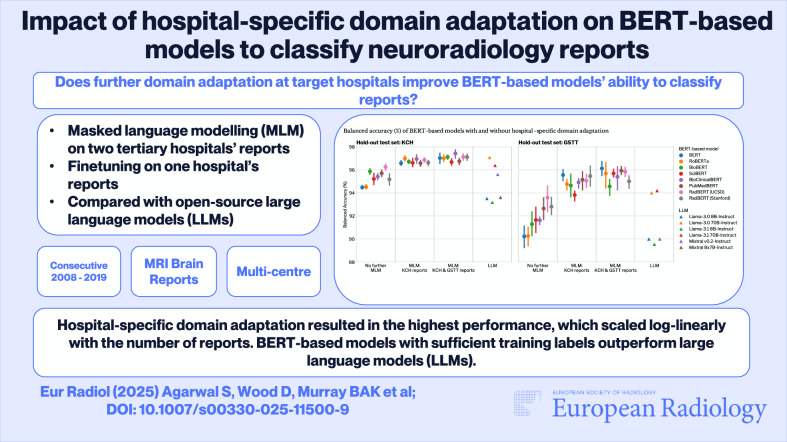

## Introduction

Artificial intelligence (AI) that performs computer vision classification tasks in medicine typically requires large amounts of training labels [1–3]. In radiology, while training labels can be created by radiologists actively viewing images, they can also be extracted retrospectively from reports [4–7]. However, this labelling process requires expert-level domain knowledge, which is a scarce and expensive resource [8, 9]. Automated methods for report classification could address this challenge whilst offering additional clinical benefits. For example, consultant radiologists could prioritise reviewing abnormal reports from on-call trainee radiologists, with multi-label classification enabling refined triage by distinguishing urgent pathologies (e.g., stroke) from less critical conditions (e.g., atrophy). Such automated extraction from large volumes of unstructured reports would also be invaluable for clinical audits, particularly for identifying relevant subsets (e.g., identifying a subset of reports with stroke, for an audit of adherence to stroke imaging protocols); the same information would provide training labels for AI image classification models. Across these applications, accuracy is paramount: in triage, false negatives could delay the review of abnormal findings, whilst, in image classification, mislabelled data could compromise the model’s ability to distinguish normal from abnormal findings.

One solution is for language models such as ‘bidirectional encoder representations from transformers’ (BERT) to automate report labelling. Once trained on numerous examples, BERT can generate labels for large report datasets efficiently [10]. BERT originally achieved success in classifying text in the general domain through pretraining on large, publicly available text corpora using ‘masked language modelling’ (MLM) [11]. By correctly predicting occluded words in a given sentence, BERT developed knowledge of English grammar, syntax and semantic relationships (Supplementary Material 1).

To perform a downstream task such as classifying reports as ‘normal’ or ‘abnormal’, pretrained models need to be ‘fine-tuned’ with labels. This transfer learning approach leverages the weights learnt through MLM, alongside a final layer designed for classification. Since MLM is self-supervised it requires no labels, but supervised fine-tuning requires manual labels for the model to learn from.

As an intermediate step, MLM can be continued on a different body of text for domain adaptation. Continuing MLM on an in-domain corpus before fine-tuning often results in better performance than fine-tuning alone. BioBERT, for example, was initialised from BERT and further domain-adapted to scientific literature with MLM, thereby developing a knowledge of ‘scientific English’ [12]. Similarly, for domain adaptation to radiology reports, RadBERT models have been further pretrained with MLM on over 4 million radiology reports with greater downstream fine-tuned performance on radiology-specific tasks (e.g., abnormality classification) than the models they were initialised from [13, 14]. When adapting to a new radiology task at a different hospital, it is currently unclear whether another intermediate step, specifically further hospital-specific domain adaptation of specialised models such as RadBERT, yields additional performance gains.

The purpose of this study was to determine the best training strategy for BERT-based models for two downstream tasks: (1) binary classification of MRI brain reports as normal or abnormal, and (2) multi-label classification assigning MRI brain reports to one or more of seven abnormality categories. The primary objective was to determine whether there was any incremental benefit in performance from additional hospital-specific domain adaptation. A secondary objective was to compare BERT-based models to open-source, state-of-the-art large language models (LLMs) [15, 16].

## Methods

The UK National Health Research Authority/Research Ethics Committee (REC) approved this retrospective methodological study (Integrated Research Application System (IRAS) ID 235,658, REC ID 18/YH/0458). We adhered to the checklist for artificial intelligence in medical imaging (CLAIM) [17].

### Data and reference standard labelling (Fig. 1a)

The dataset consisted of 126,556 and 86,032 consecutive de-identified radiology reports produced by expert neuroradiologists (UK consultant; US attending equivalent) between 2008 and 2019. These datasets contain all adult (> 18 years) MRI head examinations performed at Kings College Hospital (KCH), and Guy’s and St Thomas’ Trust Hospital (GSTT), respectively. Both hospitals are neuroscience tertiary referral centres in London, UK.

**Fig. 1:**
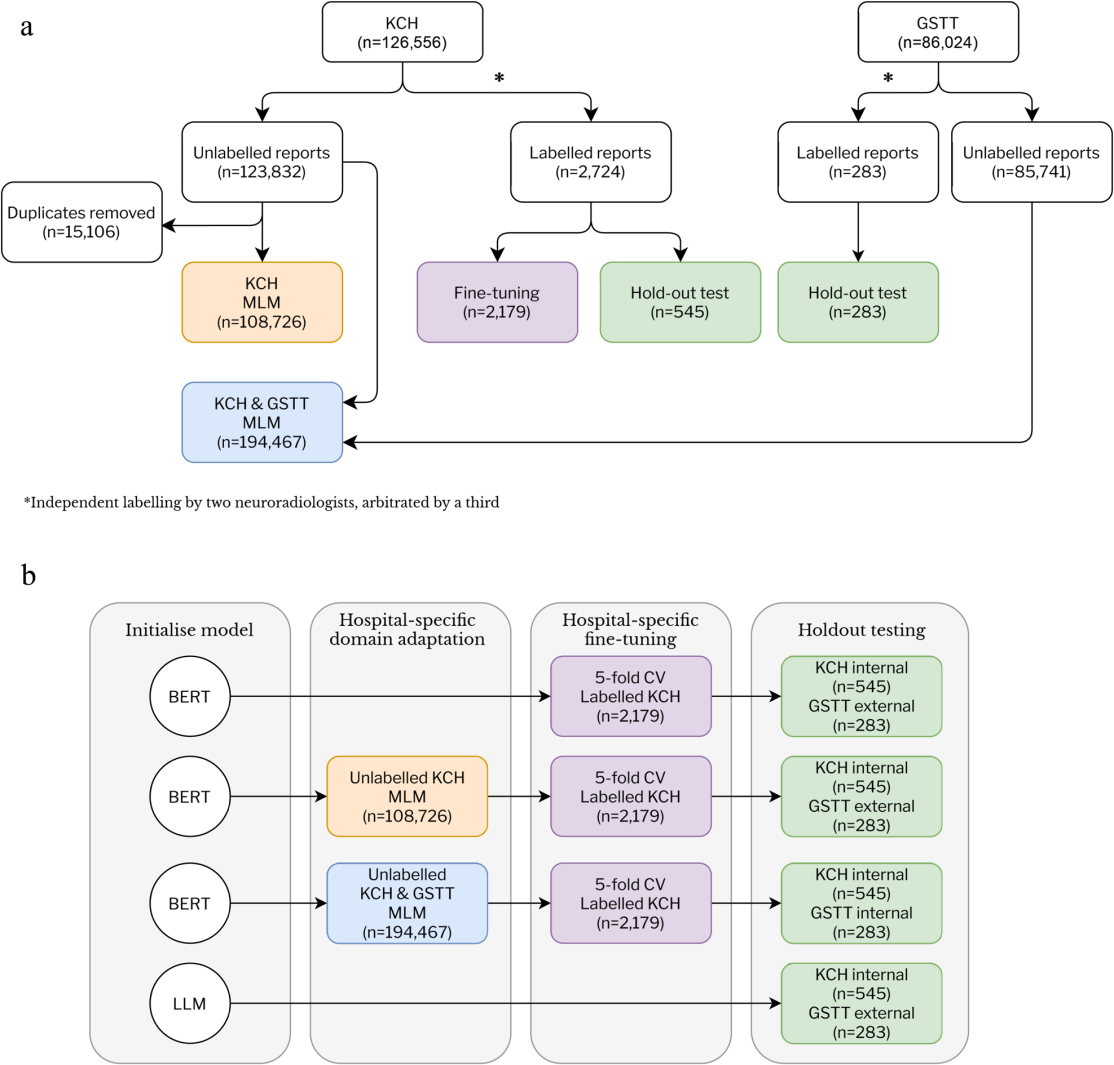
**a** Flow diagram of all adult MRI brain reports from KCH and GSTT, between 2008 and 2018. We used all adult MRI brain reports from Kings College Hospital (KCH) and GSTT between 2008 and 2018. Duplicates in the KCH dataset were removed to make MLM more computationally efficient and comprised mainly of reports stating that the MRI scan was for research purposes only. No other pre-processing of reports was done. **b** Overview of the experimental setup. Each published pretrained BERT-based model was fine-tuned with 5-fold cross-validation to determine whether brain MRIs were reported as normal or abnormal. Each published pretrained BERT-based model underwent either an additional hospital-specific domain-adaptation step by MLM (rows 2 and 3), or not (row 1). In comparison to fine-tuning which requires manual labels and is resource intensive, the hospital-specific domain adaptation step uses unlabelled data and minimal additional resource is needed. The primary objective was to determine whether there was any incremental benefit in performance from additional hospital-specific domain adaptation. We simulate data being available from a single site (row 2) or multiple sites (row 3) as this experiment would determine whether there is a benefit in collecting data from additional sites for domain adaptation. To simulate only one site with the resource to label, fine-tuning was performed using labels from one site only (column 3). Open-source LLMs were used as a benchmark comparator for the re-engineered BERT-based model performance (row 4), using a 6-shot chain-of-thought prompt. The secondary objective was to compare BERT-based models to open-source, state-of-the-art LLMs. BERT, bidirectional encoder representations from transformers; LLM, large language model; MLM, masked language modelling; KCH, King’s College Hospital; GSTT, Guys & St Thomas’ Trust; CV, cross-validation

### Report pre-processing

We randomly sampled over 1000 reports to verify the absence of patient-identifiable information. Prior to any experiments, we only kept fields for report text and training labels (‘normal’ or ‘abnormal’), omitting all other fields including hospital ID, date of birth, and sex. The report text, which typically included the clinical referral from the requesting clinician, was not pre-processed to preserve all potentially relevant information.

For computational efficiency, we removed 15,106 duplicate reports from the unlabelled King’s College Hospital (KCH) report corpus using a simple string-matching approach in Python. This deduplication process removed reports with identical text content, regardless of their clinical significance. Duplicates often included research study notes, reports with no text, or normal reports lacking clinical details. However, reports with identical findings but different clinical information in the referral text were retained as separate entries. The unlabelled Guys & St Thomas’ Trust Hospital (GSTT) report corpus contained no duplicates.

### Reference standard for task 1: binary abnormality classification

2724 reports from KCH and 283 reports from GSTT were randomly selected with an arbitrary 10:1 ratio to be labelled as ‘abnormal’ or ‘normal’. These reports have previously been used for the same classification task in a previous study, but without hospital-specific domain adaptation [4]. Over six months, two expert neuroradiologists (A.A.B. and S.K., 15 years and 16 years of neuroradiology experience, respectively) independently labelled the reports using criteria that underwent five rounds of expert panel refinement (Supplementary Material 2 defines clinically relevant abnormalities). The boundary between ‘normal’ and ‘abnormal’ was not trivial—for example, abnormal small vessel disease was defined as Fazekas II or higher, while abnormal atrophy was defined as “volume loss in excess of age”. The initial agreement rate between the two labellers was 94.9%. Disagreements were arbitrated by a third neuroradiologist (T.C.B., 19 years of neuroradiology experience) to reach a consensus.

We randomly split the labelled KCH reports into two groups, whilst maintaining consistent abnormal-to-normal label ratios: 20% (545/2724) as an ‘internal’ hold-out test set, and 80% (2179/2724) as fine-tuning training data for abnormality detection [18]. All GSTT reports (283/283) were used as an ‘external’ hold-out test set. We ensured no data leakage in any test set.

### Reference standard for task 2: multi-label ‘granular’ category classification

684 reports from KCH were randomly selected. Two expert neuroradiologists (A.A.B. and S.K.) independently labelled the presence or absence of pathology across seven predefined classes of brain abnormalities: white matter inflammation, mass, atrophy, vascular abnormalities, acute stroke, small vessel disease, and encephalomalacia (all defined in Supplementary Material 2). Although each report could be described as containing zero, one or multiple abnormality categories, our modelling approach was strictly multi-label classification, where each of the seven categories was predicted independently (present vs absent). A single report could be positive for multiple categories simultaneously (e.g., both ‘mass’ and ‘vascular’). This ‘granular’ subset was randomly split into two groups: 20% (137/684) as a hold-out test set, and 80% (547/684) as fine-tuning training data. We ensured no data leakage in any test set.

### Experimental setup (Fig. 1b)

For each BERT-based model listed in Table 1, we created three variants:

1. Baseline (Fig. 1b, row 1): the published BERT-based model, pretrained on other text corpora (Table 1).
2. Single-site domain adaptation (Fig. 1b, row 2): BERT-based model trained with MLM on unlabelled KCH reports.
3. Multi-site domain adaptation (Fig. 1b, row 3): BERT-based model trained with MLM on unlabelled KCH and GSTT reports.

**Table 1:** Published BERT-based models chosen for this study, demonstrate the corpus vocabulary and size that they were pretrained on, and whether they were initialised from a previously published BERT-based model or from scratch

Model	Vocabulary	Base model weights	Text corpus for MLM	Text corpus size
BERT (base, uncased) [10]	Wikipedia, BookCorpus	From scratch	Wikipedia, BookCorpus	3.3B words (16 GB)
RoBERTa (base) [42]	Wikipedia, BookCorpus, CC-News, OpenWebText, Stories	From scratch	Wikipedia, BookCorpus, CC-News, OpenWebText, Stories	160 GB
BioBERT v1.1 [12]	Wikipedia, BookCorpus	BERT	PubMed	4.5B words
SciBERT [43]	PMC and CS, abstracts and full texts	From scratch	PMC and CS, abstracts and full texts	1.14 M academic publications, 3.2B words
Bio_ClinicalBERT [44]	Wikipedia, BookCorpus	BioBERT v1.0	MIMIC-III: clinical notes and data (including imaging reports) from critical care patients	0.5B words (3.7 GB)
PubMedBERT [40]	PubMed, abstracts and full texts	From scratch	PubMed, abstracts and full texts	3.1B words
RadBERT(UCSD) [13]	Wikipedia, BookCorpus, CC-News, OpenWebText, Stories	RoBERTa	All available radiology reports	4.42 M radiology reports
RadBERT (Stanford) [14]	Wikipedia, BookCorpus	BioBERT	All available radiology reports	4.05 M radiology reports

Many of the models have previously been used as reference standards (benchmarks) for comparison in the biomedical domain [40]. The two RadBERT models represent the most extensively pretrained BERT-based models on radiology text that are publicly available, trained on more than 4.05 million and 4.42 million reports, respectively, from all subspecialties and modalities [13, 14]. All model weights were accessed from the Hugging Face repository [41]

*BERT* bidirectional encoder representations from transformers, *RoBERTa* a robustly optimized BERT pretraining approach, *UCSD* University of California San Diego, *PMC* PubMed Central, *CS* computer science, *MIMIC* medical information mart for intensive care, *GB* gigabytes, *B* billion, *M* million

### Hospital-specific domain adaptation (Fig. 1b, column 2)

Domain adaptation was performed using MLM on unlabelled reports, where models were trained to predict words within the report that were occluded randomly (‘masked’). We masked 25–35% of whole words dynamically during training and a dynamic learning rate schedule was used to ensure convergence (see Supplementary Material 4 for hyperparameter details).

Reports were not pre-processed in any way apart from the exclusion of 15,106 duplicates in the unlabelled KCH dataset, which were excluded for computational efficiency. We did not use labelled reports from task 1 (Fig. 1a) to mitigate any concerns over data leakage, and during MLM, models from both tasks 1 and 2 had no access to any training labels.

### Fine-tuning for task 1: binary abnormality classification (Fig. 1b, column 3)

After any hospital-specific domain adaptation, we fine-tuned each model variant for binary classification (normal/abnormal) using 2179 labelled KCH reports (80% of 2724 total). We employed 5-fold cross-validation, maintaining consistent abnormal-to-normal label ratios across training and validation sets.

Fine-tuning involved training for ten epochs, and model weights were saved when the highest balanced accuracy on the validation set was achieved. Further details, including hyperparameter selection, are provided in Supplementary Material 5.

### Fine-tuning for task 2: multi-label ‘granular’ category classification

After hospital-specific domain adaptation, we fine-tuned each model variant for multi-label classification (seven abnormality classes as present/absent) using 547 labelled KCH reports for 5-fold cross-validation. Fine-tuning involved training for 30 epochs, and model weights were saved when the highest balanced accuracy on the validation set was achieved (further details in Supplementary Material 5).

### Testing for task 1: binary abnormality classification (Fig. 1b, column 4)

All five fine-tuned models for each of the three variants of each published BERT-based model were tested on hold-out test sets from both KCH and GSTT. Performance was reported as mean and standard deviation of balanced accuracies for each model variant. Balanced accuracy is defined as the arithmetic mean of sensitivity and specificity.

### Testing for task 2: multi-label ‘granular’ category classification

All five fine-tuned models (one for each cross-validation fold) per original BERT-based were tested on the hold-out test set from the KCH ‘granular’ set. During testing, each model had to simultaneously predict the presence or absence of each of the seven abnormality categories. Performance was reported as mean and standard deviation of balanced accuracies for each model variant.

### Task 1 comparison with a bag-of-words

To provide a baseline for comparison, we implemented a bag of words (BoW) model, a traditional text representation method. The BoW approach converts each MRI report into a sparse feature vector based on the presence or frequency of words, without considering their order or context. By comparing this baseline to our BERT-based models, we aim to highlight the advantages of leveraging contextualized embeddings for complex classification tasks. We used cross-validation folds for training as the BERT-based models above for Task 1. For each fold, a new BoW matrix was created from which a logistic regression classifier was fit.

### Task 1 comparison with LLMs (Fig. 1b, row 4)

We additionally compared BERT-based models to the performance of LLMs. The main advantage of LLMs in this context is that they do not require labelled data.

We evaluated six recent, state-of-the-art open-source LLMs: 8B-Instruct and 70B-instruct versions of both Llama 3.0 and Llama 3.1 from Meta, Mistral v0.2-Instruct and Mixtral 8x7B v0.1-Instruct from Mistral AI [15, 16, 19, 20]. We deployed these models locally as we wanted to avoid the privacy concerns associated with proprietary LLMs such as GPT-4 or Google Gemini [21, 22].

We iteratively refined the prompt using Mistral v0.2-Instruct on the training set of ‘fold 1’ from the labelled KCH dataset. This optimised prompt was then applied consistently across all LLMs for testing on both KCH and GSTT hold-out test sets. We used model settings that resulted in near-deterministic responses, yielding a single result per model.

The following prompt-engineering techniques were employed:

- Chain-of-thought prompting [23]: we provided intermediate reasoning steps for the model to follow.
- Context inclusion: condensed label criteria for human labourers Supplementary Material 2) was included in the prompt.
- JSON output: we requested responses in JSON format, for consistency and for ease of parsing in Python.
- Few-shot prompting [24]: we included six example reports with ideal outputs (the maximum we could fit within the token limits).

We used a Python script to automatically convert all LLM responses into a binary abnormality label. LLM responses from the hold-out test sets were not reviewed prior to reporting the final performance accuracies of each model. The specific prompt, engineering methods, and model parameters are detailed in Supplementary Material 6.

### Ablation study

To study the impact of dataset size on model performance, we reduced the number of reports available for MLM. We created subsets of varying sizes from two datasets: unlabelled KCH reports alone, and the combined unlabelled reports from KCH and GSTT. This allowed us to analyse both the effect of dataset size and the impact of including data from multiple institutions. To maintain data consistency, each report subset was derived from the next largest set (e.g., the 60% KCH subset was a subset of the 80% KCH subset, which in turn was drawn from all unlabelled KCH reports).

We chose RoBERTa as the published BERT-based model for MLM on these subsets. RoBERTa was the base model for the University of California, San Diego (UCSD)-RadBERT, which was also trained with MLM. Therefore, we could compare the performance of datasets for MLM—unlabelled KCH and GSTT MRI brain reports (in-domain) compared to RadBERT’s 4.42 million US radiology reports from all sub-specialities and modalities (related but out-of-domain). RoBERTa and UCSD-RadBERT models were fine-tuned on labelled KCH reports following MLM (column 3, Fig. 1b).

### Software

The above methods were implemented in Python [18, 22, 25–27]. We primarily used PyTorch and Hugging Face’s Transformers libraries for model training [28, 29]. Statistical analyses were conducted using the statsmodels module [30].

### Statistical testing

For binary classification, two-way analysis of variance (ANOVA) was the primary analysis to examine the effect of hospital-specific domain adaptation while accounting for differences between BERT-based models. This tested the influence of (1) MLM dataset choice (none vs KCH vs KCH and GSTT), (2) BERT-based model choice, and (3) their interaction on balanced accuracy. We report two measures of effect size: (1) the mean improvements in balanced accuracy from hospital-specific domain adaptation with MLM, with 95% confidence intervals (CI) derived from post-hoc Tukey tests and (2) standardised effect sizes with Cohen’s d [31], with 95% CI provided by the Hedges & Olkin approximation [32].

One-way ANOVA assessed whether there were any differences between BERT-based models within MLM groups (rows 1–3, Fig. 1b). For the ablation study, a separate two-way ANOVA examined the MLM dataset, number of reports, and their interaction as factors. The threshold for significance for all tests was *p* = 0.05. The results and tests of normality and homogeneity of variances are detailed in Supplementary Material 7.

## Results

### Dataset characteristics

The characteristics of patients in both KCH and GSTT datasets and their subsequent samples are summarised in Table 2.

**Table 2:** Characteristics of the reports used for MLM, fine-tuning and testing

	All KCH reports	KCH MLM set	Labelled KCH reports	KCH test set	KCH ‘granular’ set	KCH ‘granular’ test subset	All GSTT reports	GSTT MLM set	GSTT test set
Total reports	126,556	108,726	2,724	545	684	137	86,024	85,741	283
Median age (Q1–Q3)	44.9 (27.3–60.5)	47.7 (31.4–59.0)	44.9 (31.4–59)	45.0 (31.9–59.2)	53.3 (39.5–71.7)	52.3 (39.1–69.6)	49.0 (36.0–63.0)	49.0 (36.0–63.0)	48.0 (35.0–63.0)
Female patients (%)	52.6	54.7	53.4	53.0	53.2	58.3	57.8	57.7	60.1
Overall abnormality (%)	–	–	40.9	40.9	–	–	–	–	54.4
White matter inflammation (%)	–	–	–	–	25.4	25.5	–	–	–
Mass (%)	–	–	–	–	13.6	19.7	–	–	–
Atrophy (%)	–	–	–	–	32.9	32.8	–	–	–
Vascular (%)	–	–	–	–	10.2	6.6	–	–	–
Stroke (%)	–	–	–	–	7.6	3.6	–	–	–
Small vessel disease (%)	–	–	–	–	17.0	16.1	–	–	–
Encephalomalacia (%)	–	–	–	–	25.3	16.1	–	–	–

Cells were left blank where insufficient information was present to calculate. The overall abnormality for the KCH ‘granular’ set was not recorded, and the seven classes recorded do not represent all possible classes of abnormality

*MLM* masked language modelling, *KCH* King’s College Hospital, *GSTT* Guys & St Thomas’ Trust Hospital

### Model training

MLM required 23–71 h on the KCH and GSTT datasets (194,467 reports) and between 11 h and 59 h on the KCH dataset only (108,726 reports). Fine-tuning one model for binary classification took approximately 10 min, and approximately 3 min for multi-label classification. All BERT-based models required one graphics processing unit only (GPU).

LLM inference typically took less than 15 min in total. Whilst Llama-3 8B and Mistral v0.2 models only required one GPU, Mixtral 8x7B required four NVIDIA A100 (40GB) cards and Llama-3 70B models required eight in total.

### Task 1: abnormality detection performance

**Fig. 2:**
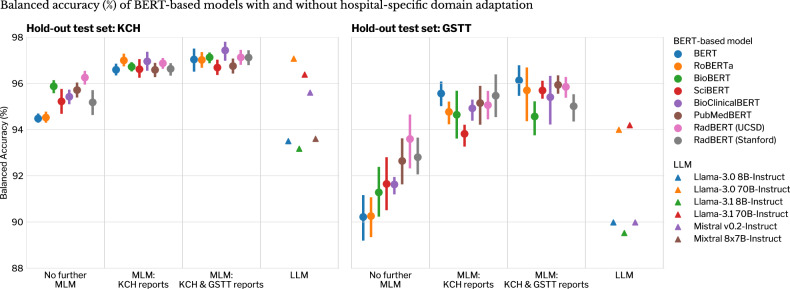
Hold-out test set performance of BERT-based models, with and without hospital-specific domain adaptation, for binary abnormality classification (determining whether an MRI brain report is normal or abnormal). For each BERT-based model, the mean balanced accuracy and 95% CI are displayed. Domain adaptation was achieved through MLM on the unlabelled reports from KCH (to simulate a single-site scenario) or the unlabelled reports from both KCH and Guys’ and St. Thomas’ Trust Hospital (GSTT) (to simulate a multi-site scenario). All fine-tuning was performed on labelled reports from KCH (to simulate a single site with the resource to label). Comparison is made with the output of open-source LLMs. BERT, bidirectional encoder representations from transformers, RoBERTa, a robustly optimized BERT pretraining approach; UCSD, University of California San Diego; KCH, King’s College Hospital; GSTT, Guys & St Thomas’ Trust Hospital; LLM, large language model; MLM, masked language modelling

Table 3 and Fig. 2 demonstrate the abnormality detection performance of all BERT-based models, with and without domain adaptation, compared with LLMs and a BoW model. Mean balanced accuracies were above 90%, regardless of domain adaptation strategy. MLM on both KCH and GSTT unlabelled reports had the highest balanced accuracies (KCH: mean 97.0 ± 0.4% (SD), GSTT: 95.5 ± 1.0%).

**Table 3:** Performance of BERT-based models and LLMs for binary abnormality classification of brain MRI reports

	KCH hold-out test set			GSTT hold-out test set		
Published BERT-based model	Variant 1 baseline model	Variant 2 MLM single site (KCH)	Variant 3 MLM multi-site (KCH & GSTT)	Variant 1 baseline model	Variant 2 MLM single site (KCH)	Variant 3 MLM multi-site (KCH & GSTT)
BERT (base)	94.48 ± 0.18	96.59 ± 0.26	97.04 ± 0.56	90.22 ± 1.26	**95.56** ± 0.62	**96.13** ± 0.77
RoBERTa (base)	94.52 ± 0.23	**96.99** ± 0.28	97.02 ± 0.38	90.25 ± 1.05	94.77 ± 0.57	95.70 ± 1.52
BioBERT v1.1	95.87 ± 0.30	96.72 ± 0.20	97.13 ± 0.24	91.28 ± 1.31	94.64 ± 1.26	94.57 ± 0.89
SciBERT	95.22 ± 0.65	96.61 ± 0.45	96.69 ± 0.36	91.65 ± 1.46	93.82 ± 0.52	95.69 ± 0.43
BioClinicalBERT	95.42 ± 0.32	96.95 ± 0.47	**97.43** ± 0.44	91.62 ± 0.42	94.92 ± 0.52	95.40 ± 1.33
PubMedBERT	95.71 ± 0.36	96.59 ± 0.35	96.75 ± 0.35	92.64 ± 1.21	95.15 ± 1.02	95.94 ± 0.45
UCSD RadBERT	**96.25** ± 0.32	96.87 ± 0.24	97.13 ± 0.36	**93.59** ± 1.41	95.06 ± 0.76	95.85 ± 0.49
Stanford RadBERT	95.18 ± 0.61	96.63 ± 0.28	97.12 ± 0.35	92.80 ± 0.94	95.47 ± 1.12	95.01 ± 0.70
Overall mean ± overall standard deviation (%)	95.33 ± 0.69	96.75 ± 0.33	97.04 ± 0.41	91.76 ± 1.56	94.92 ± 0.92	95.54 ± 0.96
Bag-of-words model with logistic regression classifier	92.62 ± 0.69			92.30 ± 0.85		
LLM^*^						
Llama-3.0 8B-Instruct	93.50			89.98		
Llama-3.0 70B-Instruct	97.07			93.99		
Llama-3.1 8B-Instruct	93.17			89.52		
Llama-3.1 70B-Instruct	96.38			94.19		
Mistral v0.2-Instruct	95.60			89.98		
Mixtral 8x7B-Instruct	93.60			86.48		

Data are mean balanced accuracies ± standard deviation (%) for BERT-based models. The overall mean and standard deviation were calculated from a combined list of all individual results from each BERT-based model. Three variants of each published BERT-based model are compared: baseline (no domain adaptation), single-site domain adaptation (using King’s College Hospital (KCH) data), multi-site domain adaptation (using KCH, and Guys’ and St. Thomas’ Trust Hospital (GSTT) data). Results in bold represent the best model performance within an MLM variant. Domain adaptation was performed through MLM on unlabelled reports. All models were fine-tuned on labelled KCH reports (simulating a single site with the resource to label). For our secondary objective where we compared LLMs, we used model settings that resulted in near-deterministic responses, yielding a single result per model. LLMs were tested once per test set, therefore one value for balanced accuracy (%) is presented. Additional metrics available in Supplementary Material 8

*MLM* masked language modelling, *BERT* bidirectional encoder representations from transformers, *RoBERTa* a robustly optimized BERT pretraining approach, *UCSD* University of California San Diego, *KCH* King’s College Hospital, *GSTT* Guys & St Thomas’ Trust Hospital, *LLM* large language model

^*^ LLM outputs were deterministic—multiple outputs would yield a standard deviation of zero

Every model that underwent a hospital-specific domain adaptation step with MLM, either on single or multi-site unlabelled reports (i.e., KCH reports or combined KCH and GSTT reports), performed better at binary classification than the base model (Table 3 and Fig. 2).

Mean improvements in balanced accuracy from MLM on the KCH dataset were 1.41% (95% CI: 1.14–1.68, *p* < 0.001) and 3.17% (95% CI: 2.54–3.79, *p* < 0.001) for KCH and GSTT hold-outs respectively. In standardised terms, these improvements corresponded to large effect sizes (Cohen’s d for KCH hold-out = 1.64, 95% CI: 1.13–2.15; Cohen’s d for GSTT hold-out = 1.51, 95% CI: 1.01–2.00).

For MLM on the combined KCH and GSTT dataset the mean improvements were 1.70% (95% CI: 1.43–1.97, *p* < 0.001) and 3.78% (95% CI: 3.15–4.41, *p* < 0.001) for KCH and GSTT hold-outs respectively (Supplementary Table 4), again reflecting large effect sizes (Cohen’s d for KCH hold-out = 2.22, 95% CI: 1.66–2.78; Cohen’s d for GSTT hold-out = 2.48, 95% CI: 1.90–3.07).

A smaller improvement from MLM on the combined KCH and GSTT dataset over the KCH-only dataset was observed, with balanced accuracy increasing by 0.29% (95% CI: 0.02–0.56, *p* = 0.030; Cohen’s *d* = 0.93, 95% CI: 0.46–1.39) and 0.61% (95% CI: −0.01 to 1.24, *p* = 0.057; Cohen’s *d* = 0.71, 95% CI: 0.26–1.16) for KCH and GSTT hold-outs respectively. Differences between BERT-based models were only present without any hospital-specific domain adaptation (KCH hold-out: *F*-statistic 11.92, *p* < 0.001, GSTT hold-out: *F*-statistic 5.26, *p* < 0.001; one-way ANOVA, Supplementary Table 5). Without hospital-specific domain adaptation, biomedical and radiology BERT-based models (e.g., BioBERT, RadBERT) performed better than general models (i.e., BERT, RoBERTa). The base model with the highest means balanced accuracy was UCSD-RadBERT (KCH hold-out: 96.3%, GSTT hold-out: 93.6%). No differences between models remained after MLM with either the KCH (KCH hold-out: *F*-statistic 1.34, *p* = 0.263, GSTT hold-out: *F*-statistic 2.11, *p* = 0.071) or KCH and GSTT dataset (KCH hold-out: *F*-statistic 1.81, *p* = 0.121, GSTT hold-out: *F*-statistic 1.65, *p* = 0.159). It is noteworthy that there was no advantage of using a biomedical or radiology BERT-based model compared to general models.

On both hold-out test sets, LLMs also had high-performance accuracy. However, with the exception of Llama-3.0 70B, they appeared to be outperformed by all domain-adapted BERT-based models. Their performance was lower on the external hold-out test set compared to the internal set. In the single-site scenario where only KCH reports are available, Llama-3.0 70B appeared to outperform BERT-based models that underwent MLM on unlabelled KCH reports. In the multi-site scenario, Llama-3.0 70B performed similarly on the internal hold-out test set (97.1%), but lower on the external hold-out test set (94.0%) when compared to BERT-based models that underwent MLM on unlabelled KCH and GSTT reports. In comparison, the BoW model was the least performant model on the KCH hold-out test set but demonstrated good generalisability to the GSTT hold-out test set, where it was equivalent to BERT-based models that had not undergone hospital-specific domain adaptation.

### Ablation study for abnormality binary classification

**Fig. 3:**
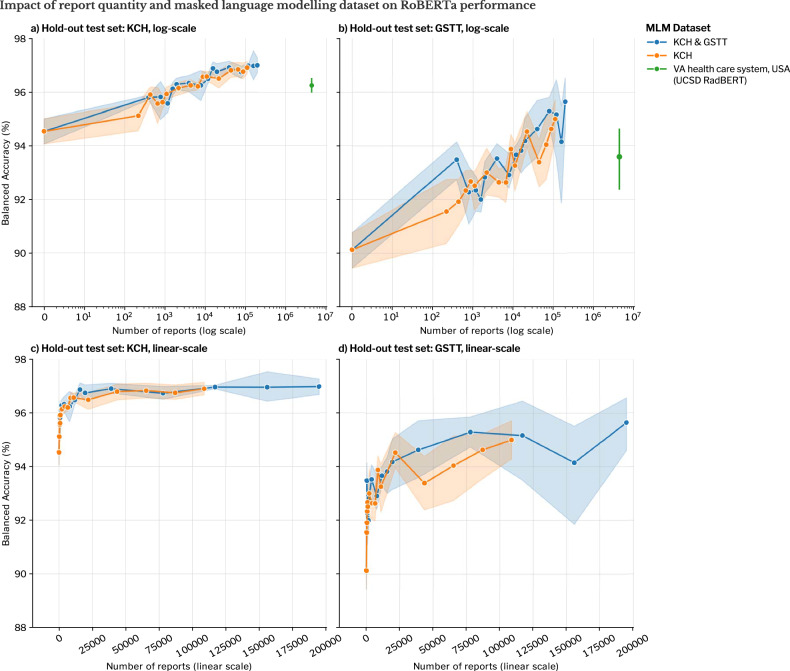
Hold-out test set performance for RoBERTa (a published BERT-based model) in binary abnormality classification as a function of the number of reports available for MLM, and the source of those reports. We measured model performance on hold-out test sets from KCH (**a**, **c**) and GSTT (**b**, **d**). Performance is displayed on a logarithmic scale (**a**, **b**) and on a linear scale (**c**, **d**). Mean balanced accuracies are displayed with the shaded bands and error bars representing the 95% CI. Domain adaptation was achieved with MLM using unlabelled MRI brain reports from either KCH (single-site scenario) or both KCH and Guys’ and St. Thomas’ Trust Hospital (GSTT) (multi-site scenario); both are tertiary neuroradiology centres in London. All fine-tuning was conducted on labelled reports from KCH. The performance is compared to UCSD RadBERT, where RoBERTa was trained with MLM on 4.42 million radiology reports from the Veterans Affairs healthcare system (USA), from all subspecialties and modalities. VA, United States Department of Veterans Affairs; UCSD, University of California San Diego; BERT, bidirectional encoder representations from transformers; RoBERTa, a robustly optimized BERT pretraining approach; KCH, King’s College Hospital; GSTT, Guys & St Thomas’ Trust Hospital

The effect of the choice and size of the dataset used for MLM is summarised in Fig. 3. For both MLM datasets, there was a log-linear relationship between the number of reports and the downstream fine-tuned balanced accuracy. The number of reports (transformed on a logarithmic scale) accounted for most of the variance in performance (KCH hold-out *F*-statistic 327.57, *p* < 0.001; GSTT hold-out *F*-statistic 176.53, *p* < 0.001; two-way ANOVA, Supplementary Table 8) rather than any differences between the KCH, and combined KCH and GSTT datasets (KCH hold-out: *F*-statistic 1.38, *p* = 0.243, GSTT hold-out: *F*-statistic 2.17, *p* = 0.142) or the interaction between these two factors (KCH hold-out: *F*-statistic 0.138, *p* = 0.711, GSTT hold-out: *F*-statistic 0.14, *p* = 0.702).

MLM with even a small number of unlabelled reports (1000) appeared to improve the fine-tuned performance of RoBERTa over the base model. We also compared the performance of RoBERTa models that underwent MLM on varying numbers of KCH and GSTT reports with the fine-tuned performance of UCSD-RadBERT (Fig. 3). Because both models are the result of applying MLM to the base RoBERTa model, we could compare the effect of MLM with an in-domain corpus (MRI brain reports written in London) with an out-of-domain corpus (RadBERT was created by MLM with US radiology reports from all subspecialties and modalities). To achieve the same mean balanced accuracy with 4.42 million out-of-domain reports, one would require 5633 and 12,267 in-domain KCH reports for the KCH hold-out test set and GSTT hold-out test set, respectively (two-way ANOVA best line fit).

**Fig. 4:**
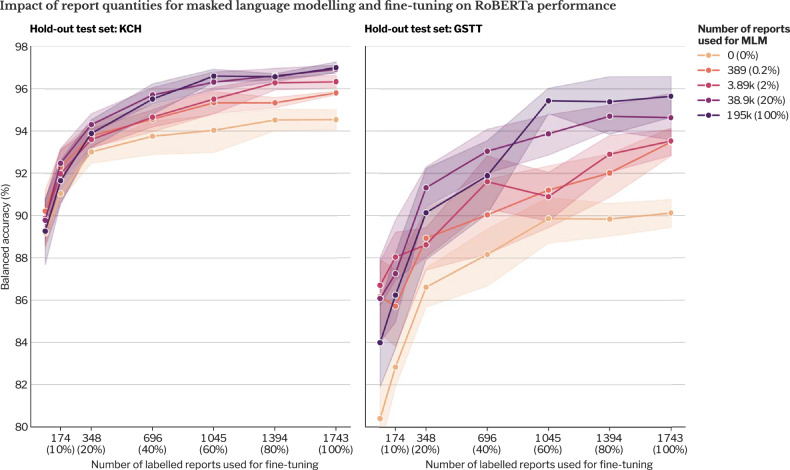
Hold out test set performance for RoBERTa (a published BERT-based model) for binary abnormality classification (determining whether a brain MRI is reported as normal or abnormal) as a function of fine-tuning dataset magnitude (number of labelled reports). Mean balanced accuracies are displayed with the shaded bands representing the 95% CI. We used the RoBERTa variant built using MLM on the unlabelled reports from both KCH and Guys’ and St. Thomas’ Trust Hospital (GSTT) (to simulate a multi-site scenario). All fine-tuning was performed on labelled reports from KCH (to simulate one site with the resource to label). RoBERTa, a robustly optimized BERT pretraining approach; KCH, King’s College Hospital; GSTT, Guys & St Thomas’ Trust Hospital

Obtaining neuroradiologist-generated training labels for fine-tuning is far more costly than the process of MLM. We therefore also investigated the effect on performance when the size of fine-tuning dataset varied (Fig. 4), where we observed that the performance of RoBERTa appeared to plateau at 1400 fine-tuning training labels.

### Task 2: multi-label ‘granular’ category classification

We extended our evaluation to a multi-label classification task, where each model simultaneously predicted the presence or absence of seven specific brain abnormality categories within MRI reports. The models assessed included various published BERT-based architectures, each tested in two variants: a baseline model without hospital-specific domain adaptation and a model with multi-site domain adaptation through MLM on unlabelled reports from both KCH and GSTT.

As shown in Table 4, models with hospital-specific domain adaptation consistently outperformed their baseline counterparts across all abnormality categories. Among these, ‘vascular’ and ‘mass’ lesions emerged as the most challenging to classify. This may be attributed to the heterogeneity of pathologies within these categories, as well as the specific ways in which they are defined (Supplementary Material 2). These findings suggest that hospital-specific domain adaptation may help with challenges arising from both complex pathology and centre-specific diagnostic criteria. These results should however be interpreted with caution as only 547 reports were used for model fine-tuning, and it is unlikely that these models achieved performance saturation.

**Table 4:** Performance of BERT-based models for multi-label classification of brain MRI reports

Published BERT-based model	Hospital-specific domain adaptation	White matter inflammation (%)	Mass (%)	Atrophy (%)	Vascular (%)	Acute stroke (%)	Small vessel disease ≥ Fazekas II (%)	Encephalo-malacia (%)
BERT (base)	None	86.76 ± 1.09	68.90 ± 2.12	91.93 ± 0.84	55.93 ± 2.27	85.04 ± 8.35	78.41 ± 4.20	82.95 ± 1.98
	Both sites	90.12 ± 0.48	**87.93** ± **1.22**	94.82 ± 1.11	77.58 ± 4.18	98.70 ± 0.48	91.88 ± 1.87	93.70 ± 1.39
RoBERTa (base)	None	86.93 ± 0.97	81.29 ± 4.99	91.88 ± 1.97	69.44 ± 5.32	79.22 ± 7.24	78.13 ± 6.25	86.76 ± 7.38
	Both sites	90.06 ± 1.04	83.53 ± 3.43	92.79 ± 1.03	75.01 ± 4.36	98.35 ± 0.75	91.08 ± 0.82	93.35 ± 1.25
BioBERT v1.1	None	86.71 ± 1.88	85.32 ± 2.44	91.34 ± 2.65	73.12 ± 1.83	97.57 ± 0.98	86.20 ± 6.39	92.11 ± 1.48
	Both sites	90.47 ± 0.33	85.34 ± 2.12	94.38 ± 1.07	76.82 ± 3.66	**99.39** ± **0.21**	91.32 ± 2.63	92.68 ± 2.35
SciBERT	None	88.09 ± 1.31	84.75 ± 1.85	91.64 ± 1.11	61.42 ± 3.80	86.09 ± 4.60	79.45 ± 3.48	88.09 ± 3.31
	Both sites	90.36 ± 0.62	85.06 ± 1.17	93.35 ± 0.65	**78.02** ± **4.41**	99.13 ± 0.48	**93.22** ± **1.88**	90.95 ± 1.66
BioClinical-BERT	None	87.63 ± 1.59	83.40 ± 1.63	89.47 ± 4.45	69.02 ± 9.37	87.91 ± 0.93	72.00 ± 7.30	88.37 ± 2.30
	Both sites	90.84 ± 0.74	85.80 ± 1.67	93.35 ± 0.97	75.80 ± 6.72	99.13 ± 0.48	92.31 ± 1.38	94.13 ± 1.03
PubMed-BERT	None	89.14 ± 1.31	86.14 ± 2.53	94.67 ± 0.85	65.78 ± 4.72	92.00 ± 5.35	86.18 ± 2.18	94.36 ± 0.97
	Both sites	**91.45** ± **0.52**	87.52 ± 1.83	**95.29** ± **0.59**	73.07 ± 2.14	99.74 ± 0.21	92.92 ± 2.14	**95.65** ± **0.84**
UCSD RadBERT	None	89.07 ± 0.53	81.18 ± 2.08	93.56 ± 1.88	71.00 ± 6.91	88.78 ± 0.51	85.15 ± 3.56	94.54 ± 1.23
	Both sites	89.52 ± 1.32	84.75 ± 0.96	93.73 ± 1.60	71.42 ± 4.02	98.17 ± 0.33	90.36 ± 3.10	95.15 ± 0.74
Stanford RadBERT	None	86.42 ± 2.22	80.16 ± 3.36	88.01 ± 1.75	60.90 ± 2.06	96.87 ± 3.66	72.57 ± 1.98	83.47 ± 3.56
	Both sites	90.07 ± 1.38	84.40 ± 2.09	94.11 ± 0.93	71.44 ± 4.80	97.22 ± 4.07	91.49 ± 1.82	93.31 ± 0.74

Data are mean balanced accuracies ± standard deviation (%). The overall mean and standard deviation were calculated from a combined list of all individual results from each cross-validation fold. Two variants of each published BERT-based model are compared: baseline (no domain adaptation) and multi-site domain adaptation (using KCH, and Guys’ and St. Thomas’ Trust Hospital (GSTT) data). Results in bold represent the best model performance within an abnormality category. Domain adaptation was performed through MLM on unlabelled reports. All models were fine-tuned on KCH reports labelled for the presence or absence of seven abnormality categories (‘KCH granular set’). Additional metrics available in Supplementary Material 8

*MLM* masked language modelling, *BERT* bidirectional encoder representations from transformers, *RoBERTa* a robustly optimized BERT pretraining approach, *UCSD* University of California San Diego

Each BERT-based model had to simultaneously predict the presence or absence of each abnormality category

## Discussion

### Summary of findings

Hospital-specific domain adaptation is the additional, intermediate step of MLM on the target domain—in our case, MRI brain reports from tertiary UK neuroradiology centres.

For binary abnormality classification, MLM using all available unlabelled reports (194,467) yielded the highest balanced accuracies, with each BERT-based model better than its base model. There was no performance accuracy difference between BERT-based models following MLM. For multi-label abnormality category classification, fine-tuning on even a small number of reports (547) resulted in consistent performance improvements for each abnormality category, particularly the ‘vascular’ and ‘mass’ categories.

After controlling for the base model (RoBERTa), language modelling method, and fine-tuning for binary abnormality detection, we observed that MLM with 194,467 local MRI brain reports yielded better performance compared to 4.42 million US general radiology reports; fewer than 15,000 local MRI brain reports were sufficient to achieve comparable performance.

Without hospital-specific domain adaptation through MLM, the best performing BERT-based model for binary abnormality classification appeared to be RadBERT (UCSD), likely because the domain it trained on—4.42 million radiology reports—resembled our target domain the closest.

Llama-3.0 70B was the best performing LLM, with internal hold-out test set performance matching BERT-based models trained with MLM on 194,467 hospital-specific reports, but with slightly lower performance accuracy on the external test set.

### Meaning of the study

This study highlights two crucial insights for deploying BERT-based models in clinical settings. First, it demonstrates the importance of an often-overlooked step [4, 5, 13, 14]: performing additional domain adaptation on hospital-specific data prior to fine-tuning. This step consistently improves model performance, with as few as 1000 reports yielding benefits, and performance scaling log-linearly as more reports are used. This should be considered a best practice, even when fine-tuning from a BERT-based model that closely matches the target domain. Second, our findings challenge the conventional wisdom that more diverse, heterogeneous pretraining data inherently leads to richer, more useful representations. While models like RadBERT leverage massive, diverse radiology corpora for MLM, our results suggest that these heterogeneous representations may not provide significant advantages for hospital-specific tasks. Counterintuitively, hospital-specific domain adaptation through MLM can render prior attempts at domain adaptation redundant, emphasising the importance of tailoring models to their specific deployment environment.

### Implications for clinical deployment

The primary advantage of open-source LLMs was their strong performance without additional language modelling or fine-tuning, relying solely on a prompt that included the report to be analysed along with six labelled examples. Llama 3.0 70B was the most performant, however it required eight A100 (40GB) GPUs for inference (i.e., for each report, given a prompt). Given its size, domain adaptation through continued language modelling or fine-tuning was infeasible with the resources we had available.

LLM deployment faced two main challenges. First, developing an effective prompt proved non-intuitive and required extensive experimentation; this is an active research field [33–36]. Using Mistral v0.2-Instruct as a development model, we iteratively refined prompts using various techniques (e.g., few-shot, chain-of-thought reasoning), improving validation set balanced accuracy from 86.4% to 94.9%. When applied to Llama 3.0 70B, our optimal prompt achieved a validation set balanced accuracy of 96.6%, though minor phrasing changes reduced this by up to 2.3%—to us this represented a clinically significant variation that could affect reliability in practice. Second, while LLMs required fewer labelled examples for prompting, we still needed 436 validation reports, 545 internal test reports and 283 external test reports to verify reliability.

All BERT-based models, following hospital-specific domain adaptation, consistently outperformed Llama 3.0 70B on the external test set. The initial cost of using BERT-based models was MLM, where performance was shown to scale log-linearly with the number of reports. This was a one-off cost—with 194,467 reports, this took a median of 33 h with one GPU (< 16GB). Subsequent inference was fast and required one GPU only (< 12GB). While BERT-based models required additional labelled training data (we used 1743 reports, but observed diminishing returns above 1400), they offered a key advantage: fine-tuning followed an established deep learning pipeline where the model learnt to map text to labels independently. This eliminated the need for prompt engineering expertise or detailed task-specific knowledge. While BERT-based model fine-tuning required initial setup of the training pipeline, this was a one-time investment following established practices that could be applied to further downstream tasks, whereas LLM prompt engineering would require ongoing iterative refinement and domain expertise for each new application.

### Comparison with other studies

The closest study to ours is Huemann et al, who perform an intermediate step of language modelling on the same dataset they fine-tune on, in order to extract Deauville scores from PET/CT report [37]. Similarly, they show consistent incremental benefits from target-domain language modelling (2.0–4.4%). We were able to language model with an order of magnitude more reports and demonstrated a log-linear relationship between performance and the report volume used. Additionally, we compared more BERT-based models and, unlike other studies, state-of-the-art open-source LLMs.

It is common practice to find pretrained BERT-based models that best match the target domain, and fine-tune them with any available training labels [4, 5, 38]. It is possible that these studies may have found incremental performance benefits from additional language modelling on their datasets prior to fine-tuning. The seminal RadBERT models were language-modelled on over 4 million reports each in order to bridge the gap between the general English language domain and radiology reports [13, 14]. Yet in their own experiments, they fine-tuned on datasets whose reports did not appear in their MLM dataset; for example, both studies used chest radiograph reports from separate datasets in classification tasks. Plausibly, they could also have achieved incremental benefit from additional MLM on chest radiograph reports from the same dataset that they fine-tuned.

### Limitations

We demonstrated BERT-based model performance in only one downstream task of binary classification of brain MRI reports. With additional high-quality training labels, further downstream tasks would determine the applicability of the observations in this study to other tasks.

Whilst the prompt used to generate labels from open-source LLMs was iteratively improved, it may not be the optimal prompt given the evolving in-context learning methodology evidence. Llama 3 models represent the most capable open-source LLMs to date and are superior to GPT3.5 on public benchmarks, but we were unable to use some proprietary LLMs such as GPT-4 due to privacy concerns [21, 39]. The main advantages of using open-source models are that confidential patient data is securely kept on private servers, and that the processing is affordable.

## Conclusion

For tasks similar to brain MRI report classification, we recommend implementing hospital-specific domain adaptation of BERT-based models using the maximum feasible number of reports from the target hospital. We found LLMs empirically to be highly prompt-dependent, however, they can serve as a viable alternative if labels are scarce or unavailable.

## Supplementary information

Supplementary material

## Abbreviations

BERT: Bidirectional encoder representations from transformers
BoW: Bag of words
CLAIM: Checklist for artificial intelligence in medical imaging
GPU: Graphics processing unit
GSTT: Guy’s and St Thomas’ trust
IRAS: Integrated research application system
KCH: King’s College Hospital
LLM: Large language model
MLM: Masked language modelling
REC: Research Ethics Committee
UCSD: University of California, San Diego

## References

[1] Wood DA, Kafiabadi S, Busaidi AA et al (2022) Deep learning models for triaging hospital head MRI examinations. Medical Image Analysis 78:102391. 10.1016/j.media.2022.102391 35183876 10.1016/j.media.2022.102391

[2] Chelliah A, Wood DA, Canas LS et al (2024) Glioblastoma and radiotherapy: a multicenter AI study for survival predictions from MRI (GRASP study). Neuro Oncol 26:1138–1151. 10.1093/neuonc/noae017 38285679 10.1093/neuonc/noae017 PMC11145448

[3] Wood DA, Kafiabadi S, Busaidi AA et al (2021) Automated triaging of head MRI examinations using convolutional neural networks. In: Proceedings of the fourth conference on medical imaging with deep learning. PMLR, pp 813–841

[4] Wood DA, Kafiabadi S, Al Busaidi A et al (2022) Deep learning to automate the labelling of head MRI datasets for computer vision applications. Eur Radiol 32:725–736. 10.1007/s00330-021-08132-0 34286375 10.1007/s00330-021-08132-0 PMC8660736

[5] Tejani AS, Ng YS, Xi Y et al (2022) Performance of multiple pretrained BERT models to automate and accelerate data annotation for large datasets. Radiol Artif Intell 4:e220007. 10.1148/ryai.220007 35923377 10.1148/ryai.220007 PMC9344209

[6] Wood DA, Guilhem E, Kafiabadi S et al (2024) A self-supervised text-vision framework for automated brain abnormality detection. Preprint at 10.48550/arXiv.2405.02782

[7] Wood DA, Townend M, Guilhem E et al (2024) Optimising brain age estimation through transfer learning: a suite of pre-trained foundation models for improved performance and generalisability in a clinical setting. Hum Brain Map 45:e26625. 10.1002/hbm.26625 10.1002/hbm.26625 PMC10910262 38433665

[8] Benger M, Wood DA, Kafiabadi S et al (2023) Factors affecting the labelling accuracy of brain MRI studies relevant for deep learning abnormality detection. Front Radiol. 10.3389/fradi.2023.1251825 10.3389/fradi.2023.1251825 PMC10711054 38089643

[9] Wood DA, Kafiabadi S, Al Busaidi A et al (2020) Labelling imaging datasets on the basis of neuroradiology reports: a validation study. In: Cardoso J, Van Nguyen H, Heller N et al (eds) Interpretable and annotation-efficient learning for medical image computing. Springer International Publishing, Cham, pp 254–265

[10] Devlin J, Chang M-W, Lee K, Toutanova K (2019) BERT: pre-training of deep bidirectional transformers for language understanding. Preprint at 10.48550/arXiv.1810.04805

[11] Howard J, Ruder S (2018) Universal language model fine-tuning for text classification. In: Gurevych I, Miyao Y (eds) Proceedings of the 56th annual meeting of the association for computational linguistics, vol 1: long papers. Association for Computational Linguistics, Melbourne, pp 328–339

[12] Lee J, Yoon W, Kim S et al (2020) BioBERT: a pre-trained biomedical language representation model for biomedical text mining. Bioinformatics 36:1234–1240. 10.1093/bioinformatics/btz682 31501885 10.1093/bioinformatics/btz682 PMC7703786

[13] Yan A, McAuley J, Lu X et al (2022) RadBERT: adapting transformer-based language models to radiology. Radiol Artif Intell 4:e210258. 10.1148/ryai.210258 35923376 10.1148/ryai.210258 PMC9344353

[14] Chambon P, Cook TS, Langlotz CP (2023) Improved fine-tuning of in-domain transformer model for inferring COVID-19 presence in multi-institutional radiology reports. J Digit Imaging 36:164–177. 10.1007/s10278-022-00714-8 36323915 10.1007/s10278-022-00714-8 PMC9629758

[15] Touvron H, Martin L, Stone K et al (2023) Llama 2: open foundation and fine-tuned chat models. Preprint at 10.48550/arXiv.2307.09288

[16] GitHub-meta-llama/llama3: the official Meta Llama 3 GitHub site. https://github.com/meta-llama/llama3. Accessed 1 May 2024

[17] Tejani AS, Klontzas ME, Gatti AA et al (2024) Checklist for artificial intelligence in medical imaging (CLAIM): 2024 update Radiol Artif Intell 6:e240300. 10.1148/ryai.240300 38809149 10.1148/ryai.240300 PMC11304031

[18] Pedregosa F, Varoquaux G, Gramfort A et al (2011) Scikit-learn: machine learning in python. J Machine Learning Res 12:2825–2830

[19] Jiang AQ, Sablayrolles A, Mensch A et al (2023) Mistral 7B. Preprint at 10.48550/arXiv.2401.04088

[20] Jiang AQ, Sablayrolles A, Roux A et al (2024) Mixtral of experts. Preprint at 10.48550/arXiv.2401.04088

[21] Agarwal S, Wood D, Carpenter R et al (2024) Letter to the Editor: What are the legal and ethical considerations of submitting radiology reports to ChatGPT? Clin Radiol. 10.1016/j.crad.2024.03.017 10.1016/j.crad.2024.03.017 38724415

[22] Kwon W, Li Z, Zhuang S et al (2023) Efficient memory management for large language model serving with pagedattention. Preprint at 10.48550/arXiv.2309.06180

[23] Wei J, Wang X, Schuurmans D et al (2023) Chain-of-thought prompting elicits reasoning in large language models. Preprint at 10.48550/arXiv.2201.11903

[24] Brown T, Mann B, Ryder N et al (2020) Language models are few-shot learners. in: advances in neural information processing systems. Curran Associates, Inc., pp 1877–1901

[25] Waskom ML (2021) seaborn: statistical data visualization. J Open Source Softw 6:3021. 10.21105/joss.03021

[26] Hunter JD (2007) Matplotlib: a 2D graphics environment. Comput Sci Eng 9:90–95. 10.1109/MCSE.2007.55

[27] Akiba T, Sano S, Yanase T et al (2019) Optuna: a next-generation hyperparameter optimization framework. In: Proceedings of the 25th ACM SIGKDD international conference on knowledge discovery & data mining. Association for Computing Machinery, New York, pp 2623–2631

[28] Wolf T, Debut L, Sanh V et al (2020) HuggingFace’s transformers: state-of-the-art natural language processing. 10.18653/v1/2020.emnlp-demos.6

[29] Paszke A, Gross S, Massa F et al (2019) PyTorch: an imperative style, high-performance deep learning library

[30] Seabold S, Perktold J (2010) Statsmodels: econometric and statistical modeling with python. Austin, Texas, pp 92–96

[31] Cohen J (2013) Statistical power analysis for the behavioral sciences, 2nd edn. Routledge, New York

[32] Hedges LV, Olkin I (1985) Statistical methods for meta-analysis. Elsevier Science

[33] Schulhoff S, Ilie M, Balepur N et al (2024) The prompt report: a systematic survey of prompting techniques. Preprint at 10.48550/arXiv.2406.06608

[34] Sahoo P, Singh AK, Saha S et al (2024) A systematic survey of prompt engineering in large language models: techniques and applications. Preprint at 10.48550/arXiv.2402.07927

[35] Chen B, Zhang Z, Langrené N, Zhu S (2023) Unleashing the potential of prompt engineering in large language models: a comprehensive review. Preprint at 10.48550/arXiv.2310.14735

[36] Vatsal S, Dubey H (2024) A survey of prompt engineering methods in large language models for different NLP tasks. Preprint at 10.48550/arXiv.2407.12994

[37] Huemann Z, Lee C, Hu J et al (2023) Domain-adapted large language models for classifying nuclear medicine reports. Radiol Artif Intell 5:e220281. 10.1148/ryai.220281 38074793 10.1148/ryai.220281 PMC10698610

[38] Kim M, Ong KT, Choi S et al (2023) Natural language processing to predict isocitrate dehydrogenase genotype in diffuse glioma using MR radiology reports. Eur Radiol 33:8017–8025. 10.1007/s00330-023-10061-z 37566271 10.1007/s00330-023-10061-z

[39] OpenAI, Achiam J, Adler S et al (2024) GPT-4 technical report. Preprint at 10.48550/arXiv.23038774

[40] Gu Y, Tinn R, Cheng H et al (2021) Domain-specific language model pretraining for biomedical natural language processing. ACM Trans Comput Healthcare 3 2:23. 10.1145/3458754

[41] Hugging face (2024) The AI community building the future. https://huggingface.co/. Accessed 1 May 2024

[42] Liu Y, Ott M, Goyal N et al (2019) RoBERTa: a robustly optimized BERT pretraining approach. Preprint at 10.48550/arXiv.1907.11692

[43] Beltagy I, Lo K, Cohan A (2019) SciBERT: a pretrained language model for scientific text. In: Proceedings of the 2019 conference on empirical methods in natural language processing and the 9th International Joint Conference on natural language processing (EMNLP-IJCNLP). Scientometrics, pp 1241–1263

[44] Alsentzer E, Murphy JR, Boag W et al (2019) Publicly available clinical BERT embeddings. In: Proceedings of the 2nd clinical natural language processing workshop. Association for Computational Linguistics, Minneapolis, pp 72–78

