# Supplementary material for: Impact of hospital-specific domain adaptation on BERT-based models to classify neuroradiology reports
Source: Eur Radiol. 2025 Mar 17;35(9):5299–313. doi: 10.1007/s00330-025-11500-9 (PMC12350531; doi:10.1007/s00330-025-11500-9)
Supplement: Supplementary file 1 — Supplementary material [file 330_2025_11500_MOESM1_ESM.pdf]

Supplementary material

1. Glossary 2

2. Label criteria 5

3. Report pre-processing 12

4. Masked language modelling details 13

5. Fine-tuning details 15

6. Deploying local large language models 16

Large language models tested 16

Prompt engineering techniques 16

Final prompt 18

7. Statistical analysis 24

7.1 Effect of masked language modelling dataset and BERT-based model choice on performance 24

7.2 Ablation study: effect of language modelling dataset and number of reports on performance 27

8. Additional accuracy metrics 30

Supplementary material references 47

1. Glossary

**Model**	In AI, a function that maps inputs (e.g., text, images) to outputs (e.g., predictions, classifications). In deep learning, models typically consist of multiple layers of artificial neurons forming a neural network.
**Model training**	The process by which a model learns to make accurate predictions from data. The model is presented with examples of known inputs and outputs, adjusting itself to minimize prediction errors.
**Model weights**	The trainable parameters of a model, including weights and biases. These govern the transformations applied by each neuron and are adjusted during training to improve prediction accuracy.
**Initialising a model**	Setting the initial values for a model's weights prior to training. This can be done randomly, to train from scratch. Weights can also be loaded from a pretrained model – this technique is also known as transfer learning.
**Transfer learning**	A technique where knowledge gained from training on one task is applied to a different but related task, often involving initialising a model with weights from a pretrained model before fine-tuning.
**Pretraining**	Model training that is done prior to the intended task. Useful pretraining usually involves large, diverse datasets so that models can learn general patterns and features of data. Pretraining serves as a starting point for more specific tasks, often leading to quicker convergence and better performance.
**Fine-tuning**	Adapting a pretrained model for a specific task by training it on a smaller, task-specific dataset. This process takes advantage of useful representations of data that was learnt during pretraining.
**Language modelling**	A natural language processing (NLP) pretraining task which is typically self-supervised. Models learn to predict the probability distribution of words in a sequence. By predicting words in a given sequence, models

	are able to capture patterns in language structure, grammar, and semantics.
**Masked language modelling**	A variant of language modelling where the model is trained to predict masked (hidden) words in a sentence rather than the next word in a sequence. This approach, popularised by the original "bidirectional encoder representations from transformers" (BERT) model, allows the model to learn bidirectional context and develop a deeper understanding of language. In masked language modelling, a percentage of input tokens are randomly masked, and the model is trained to predict these masked tokens based on the surrounding context.
**Domain**	The specific area of knowledge or content type that models are trained or used in, often defined by the dataset.<br><br>For example, BookCorpus is an open-source dataset scraped from 7,000 self-published books online, spanning a range of genres including romance, science fiction, and fantasy [1]. When the original BERT model was trained on BookCorpus and Wikipedia, its training domain was general English language, given the diversity of the text sources [2]. Radiology reports represent a separate, niche domain.
**In-domain**	Data from the same or very similar domain as the one the model is being applied to.<br><br>For example, a model pretrained on radiology reports is used to classify other radiology reports. This model was pretrained on in-domain data.
**Out-of-domain**	Data from a different domain than the one the model is being applied to.<br><br>For example, a model pretrained on general English language is used to classify radiology reports. This model was pretrained on out-of-domain data.
**Target domain**	The specific domain to which a model is being applied, which is inherently in-domain.

For example, a model pretrained on a particular dataset of MRI brain reports is used to classify MRI brain reports from the same dataset.

Domain shift Differences in data between the training domain and deployment domain, often resulting in a loss of performance.

For example, a computer vision model trained on scans until 2015 may lose performance when deployed on scans performed after 2020. We can speculate that factors leading to the performance drop may include a change in scanner, scanning protocol and differences in patient populations.

2. Label criteria

For previous work [3], our team designed a complete set of categories containing clinically relevant abnormalities thereby accurately capturing the full range of pathologies (and normal variants mimicking pathologies) which present on head MRI examinations. The aim here was to try and emulate the behaviour of a neuroradiologist in the real world, whilst ensuring that findings that may generate a downstream clinical intervention for an abnormal finding are included. In our ontology, clinical intervention includes referral for case discussion at a multi-disciplinary team meeting. In some cases, for example the discovery of a developmental venous anomaly, the referring clinician may not understand the clinical relevance of the reported finding and may thus need to request clarification of clinical relevance at a multi-disciplinary team meeting. We erred on the side of sensitivity so that our labelling framework would lead to algorithms producing the safest clinical decisions.
The development process required the inspection > 1000 radiology reports by our team of experienced neuroradiologists before an exhaustive and consistent set of abnormality categories, as well as the rules by which reports were to be labelled, could be finalised. The rules and definitions recursively evolved during the course of the practice labelling experiments. These criteria were fixed prior to any labelling for the current work.
Abnormal is defined as one or more abnormalities described below. Normal is defined as no abnormality described below.

Small vessel disease
Fazekas et al [4] give a classification system for white matter lesions (WMLs) summarised as:

1. Mild - punctate WMLS: Fazekas I
2. Moderate - confluent WMLs: Fazekas II
3. Severe - extensive confluent WMLs: Fazekas III

To create a binary categorical variable from this system, if the report is described as “unsure”, “normal” or “mild” this is categorised as normal as this never requires treatment for cardiovascular risk factors. However, if there is a description of moderate or severe WMLs, the report is categorised as abnormal as these cases sometimes require treatment for cardiovascular risk factors.
Included as normal are descriptions of scattered non-specific “white matter dots” or “foci of signal abnormality” (unless a more defuse or specific pathology is implied) and small vessel disease described as “minor”, “minimal” or “modest”.

Conversely, those cases which are described as “mild to moderate”, “confluent”, or “beginning to confluence” small vessel disease are treated as abnormal.
Genetic small vessel disease, in particular Cerebral Autosomal Dominant Arteriopathy with Subcortical Infarcts and Leukoencephalopathy (CADASIL), is considered abnormal.

Mass

All the following intracranial masses are categorised as abnormal:

- Neoplasms (tumours)
    - Intra-axial including all primary and secondary neoplasms
    - Extra-axial including all primary and secondary neoplasms
        - Pituitary adenomas included
    - Lipomas included
- Tumour debulking or partial resection as this implies residual tumour (note: these are labelled as both “encephalomalacia” and “mass” abnormalities)
- Ependymal, subependymal or local meningeal enhancement (non-surgical) in the context of a history of an aggressive infiltrative tumour
- Abscess
- Cysts
    - Retro cerebellar cyst (mega cisterna magna not included)
    - Arachnoid cysts
    - Pineal cysts and choroid fissure cysts
    - Rathke cleft cysts
- Focal cortical dysplasia, nodular grey matter heterotopia, subependymal nodules and subcortical tubers
- Chronic subdural hematoma or hygroma (i.e. cerebrospinal fluid (CSF) equivalent)
- Perivascular spaces normal unless giant
- MRI examinations for stereotactic surgical planning alone may have very brief reports. In these scenarios it is typically evident from the clinical information provided that there is a mass e.g., surgical planning for glioblastoma.

Note that findings that typically may have minimal clinical relevance when confirmed by a neuroradiology expert, are included in this category e.g., arachnoid cyst. The rationale is that such a finding might generate a referral to a multidisciplinary team meeting for clarification clinical relevance. We consider that a referral to a multidisciplinary team meeting is a clinical intervention and we aim to ensure that any findings that generate a downstream clinical intervention are included.

Vascular

All the following are categorised as abnormal for vascular:

- Aneurysm
    - including coiled aneurysms regardless of whether there is a residual neck or not
- Arteriovenous malformation
- Arteriovenous dural fistula
- Cavernoma
- Capillary telangiectasia
- Chronic / non-specific microhaemorrhages
- Petechial haemorrhage
- Developmental venous anomaly
- Venous sinus thrombosis
- Vasculitis if associated with vessel changes such as luminal stenosis or vessel wall enhancement
- Arterial occlusion / flow void abnormality or absence
- Venous sinus tumour invasion (this is labelled as both “vascular” and “mass” abnormalities)
- Arterial stenosis. If constitutional / normal variant not included.
- Vascular-like findings which are considered normal include descriptions of sluggish flow, flow-related signal abnormalities (unless they raise the suspicion of thrombus) and vascular fenestrations.

Note that findings that typically may have minimal clinical relevance when confirmed by a neuroradiology expert, are included in this category e.g., developmental venous anomaly. The rationale is that such a finding might generate a referral to a multidisciplinary team meeting for clarification of clinical relevance. We consider that a referral to a multidisciplinary team meeting is a clinical intervention, and we aim to ensure that any findings that generate a downstream clinical intervention are included.

Encephalomalacia

All the following are categorised as abnormal for encephalomalacia:

- Gliosis
- Encephalomalacia
- Cavity
- Post-operative tissue changes / appearances are included as encephalomalacia
- Tumour debulking or partial resection as this implies residual tumour (note: these are labelled as both “encephalomalacia” and “mass” abnormalities)
- Chronic infarct / sequelae of infarct

- Chronic haemorrhage / sequelae of haemorrhage (with / without haemosiderin staining)
- Cortical laminar necrosis

Encephalomalacia-like findings which are considered normal unless there is a clear description of related parenchymal injury include craniotomy, burr-holes, posterior fossa decompression, and 3rd ventriculostomy

Acute stroke

All the following are categorised as abnormal for acute stroke:

- Acute / subacute infarct (if demonstrating restricted diffusion)
    - Include if there are other descriptors indicating a subacute nature such as swelling even though restricted diffusion has normalised
- If a single ischemic event with both diffusion restricting and non-restricting elements then this is labelled as an “acute stroke” abnormality (rather than an “encephalomalacia” abnormality)
- Parenchymal post-operative restricted diffusion secondary to retraction injury
    - Mitochondrial Encephalopathy with Lactic Acidosis and Stroke-like episodes (MELAS) if associated with restricted diffusion
    - Hypoxic ischemic injury if associated with restricted diffusion
    - Vasculitis if associated with acute / subacute infarct
    - “Mature”, “established”, “chronic” or “old” infarcts without other descriptors are labelled as “encephalomalacia” abnormalities

White matter inflammation

All the following are categorised as abnormal for white matter inflammation:

- Multiple sclerosis (MS) including when some plaques show cavitation (low T1 signal)
- Other demyelinating lesions including Acute Disseminated Encephalomyelitis (ADEM) and Neuromyelitis Optica spectrum disorder (NMO)
- Inflammatory lesions in Radiologically Isolated Syndrome / Clinically Isolated Syndrome
- Focal cortical thinning i.e., secondary to chronic subcortical / cortical lesions, are labelled as “encephalomalacia” abnormalities
- Progressive Multifocal Leukoencephalopathy (PML)/ Immune Reconstitution Inflammatory Syndrome (IRIS)
- Leukoencephalopathies - congenital or acquired (including toxic)
- Encephalitis / encephalopathy if it involves the white matter, e.g. related to human immunodeficiency virus (HIV) and congenital cytomegalovirus (CMV)
- Posterior Reversible Encephalopathy Syndrome (PRES)
- Osmotic demyelination (central pontine myelinolysis/ extrapontine myelinolysis)

- Susac syndrome
- Radiation if describing white matter abnormality
- White matter changes in the context of vasculitis if clearly attributed to vasculitis.
- Amyloid-related inflammatory change / inflammatory

Atrophy

Volume loss in excess of age

Hydrocephalus

- Acute
- Trapped ventricle
- Chronic / stable / improving hydrocephalus (it does not matter whether its compensated or not)
- Ventricular enlargement
- normal pressure hydrocephalus (NPH)

Haemorrhage

- Any acute / subacute haemorrhage parenchymal, subarachnoid, subdural, extradural
- Acute microhaemorrhages / petechial haemorrhages

Foreign body

- Shunts
- Clips
- Coils
- If significant metalwork is involved in skull repair e.g. in a cranioplasty (or the occasional craniotomy causing extreme intracranial MRI signal distortion)
- If craniotomies are not causing anything other than slight artefact, then these are considered normal

Extracranial

- Total mastoid opacification / middle ear effusions
- Complete opacification / obstruction of the paranasal sinuses
- Mucosal thickening is not included
- If there is clearly a well-defined unambiguous polyp then label as abnormal.
- If "retention cysts" or "polypoid mucosal thickening" then label as normal. If it is something indistinguishable which could be a retention cyst / polyp then label as normal.
- Anything leading to sinus obstruction always label as abnormal.

- Calvarial / extra-calvarial masses
- Osteo-dural defects
- Encephaloceles
- Pseudo-meningoceles
- Extracranial vascular abnormalities i.e., below the petrous segment e.g. cervical internal carotid artery (ICA) dissection
- Extracranial masses including lipoma or sebaceous cyst
- Orbital abnormalities
    - Including optic nerve pathology affecting the orbital segment of the nerve i.e., meningioma
- Cases with isolated tortuous optic nerve sheath complexes with no other features suggestive of raised intracranial pressure, are labelled as normal
- Eye prostheses and proptosis
- Pseudophakia is labelled as normal
- Bone abnormality e.g., low bone signal secondary to haemoglobinopathy
- Basilar invagination
- Hyperostosis is considered normal
- Tornwaldt cysts are considered normal

Intracranial miscellaneous

- Cerebellar ectopia
- Brain herniation (e.g., through a craniectomy defect)
- Clear evidence of intracranial hypertension (e.g. prominent optic nerve sheaths AND intrasellar subarachnoid herniation)
    - Isolated intrasellar subarachnoid herniation / empty sella is labelled normal
    - Isolated tapering of dural venous sinuses is labelled normal
- Clear evidence of intracranial hypotension (e.g., pituitary enlargement AND pachymeningeal thickening)
    - If subdural collections present, these are also labelled as “mass”
- Cerebral oedema or reduced CSF spaces from parenchymal swelling
- Absent or hypoplastic structures such as agenesis of the corpus callosum
- Meningeal thickening or enhancement for example in the context of neurosarcoid or vasculitis
- Enhancing or thickened cranial nerves
- Infective processes primarily involving the meninges or ependyma (i.e. ventriculitis or meningitis)

- Encephalitis if primarily involving the cortex (herpes simplex virus (HSV)/ autoimmune encephalitis)
- Excessive or unexpected basal ganglia or parenchymal calcification
- Optic neuritis involving the intracranial segments of the optic nerves or chiasmitis
- Adhesions / webs
- Pneumocephalus
- Colpocephaly
- Superficial siderosis
- Ulegyria
- Focal areas of signal intensity (FASIs) / Unidentified bright objects (UBO)
- Basal ganglia / thalamic changes in the context of metabolic abnormalities
- Neurovascular conflict fulfilling conditions of nerve distortion AND nerve root entry zone involvement
- Band heterotopia and polymicrogyria
- Hypophysitis
- Seizure related changes
- Amyotrophic lateral sclerosis (ALS).

3. Report pre-processing

We randomly sampled over 1,000 reports to verify the absence of patient-identifiable information. Prior to any experiments, we only kept fields for report text and training label (‘normal’ or ‘abnormal’), omitting all other fields including hospital ID, date of birth, and sex. The report text, which typically included the clinical referral from the requesting clinician, was not pre-processed to preserve all potentially relevant information.

For computational efficiency, we removed 15,106 duplicate reports from the unlabelled King’s College Hospital (KCH) report corpus using a simple string-matching approach in Python. This deduplication process removed reports with identical text content, regardless of their clinical significance. Duplicates often included research study notes, reports with no text, or normal reports lacking clinical details. However, reports with identical findings but different clinical information in the referral text were retained as separate entries. The unlabelled Guys & St Thomas’ Trust Hospital (GSTT) report corpus contained no duplicates.

Example of a research study note: MRI Functional imaging : MRI Head IOP Research scan. These contain limited sequences. Not to be used for clinical purposes. These images have not been reviewed by a Neuroradiologist, as an MRI research request form was not submitted by the researcher.

Example of a deduplicated normal report: Normal intracranial appearances.

4. Masked language modelling details

Hospital-specific domain adaptation was achieved through masked language modelling (MLM), similar to the original BERT paper [2]. We modified the approach based on more recent work [5, 6]:

- Masking whole words instead of tokens
- Dynamically altering masked words per training epoch
- Omitting 'next sentence prediction' as an additional training objective

To ensure convergence and avoid optimising the number of training epochs as an additional hyperparameter, we:

- Implemented a dynamic learning rate schedule with a 2-epoch warmup to a maximum learning rate, followed by successive halving of the rate if validation loss did not decrease within 5 epochs
- Applied early stopping, terminating training if validation loss did not decrease within 50 epochs

We used random 3% selections of the unlabelled KCH and GSTT report corpus (5,834 reports), split in a 2:1 ratio for training and validation, to find optimal hyperparameters for each BERT-based model (Supplementary Table 1). The Optuna framework was used for hyperparameter optimization [7]. We used the Adam optimizer with a weight decay of 0.01 [8].

Supplementary Table 1: Hyperparameters used for masked language modelling

Model	Batch size	Maximum learning rate	Proportion of words masked (%)
BERT (base, uncased) [9]	16	5×10^{-5}	30
RoBERTa (base) [5]	16	4×10^{-5}	35
BioBERT v1.1 [10]	16	5×10^{-5}	30
SciBERT [11]	16	2×10^{-5}	35
Bio_ClinicalBERT [12]	16	5×10^{-5}	30
PubMedBERT [13]	16	3×10^{-5}	25
RadBERT(UCSD) [14]	16	4×10^{-5}	35
RadBERT (Stanford) [15]	16	5×10^{-5}	30

BERT: Bidirectional Encoder Representations from Transformers, RoBERTa: A Robustly Optimised BERT Pretraining Approach, UCSD: University of California San Diego

5. Fine-tuning details

Each model was fine-tuned over 10 epochs, and model weights were saved when the highest balanced accuracy on the validation set was achieved. No layers were frozen during model fine-tuning. To determine optimal hyperparameters for fine-tuning for each BERT-based model:

- We used 2,179 labelled KCH reports (separate to the 545 reports held-out as a test set).
- The Optuna framework was used for hyperparameter optimization [7].
- We randomly split this set in 80:20 training-to-validation ratios for each test in an Optuna study, maintaining abnormal-to-normal label ratios.

We empirically found that altering hyperparameters made little difference in convergence or validation accuracy. Therefore, we used the following settings for all models:

- Epochs: 10
- Learning rate scheduler: warmup over 1% of total training steps, followed by linear decay to 0 over 10 epochs
- Maximum learning rate: 2×10^{-5}
- Batch size: 8
- Optimizer: Adam with a weight decay of 0.01 [8].

6. Deploying local large language models

Large language models tested

We used the VLLM Python library [16] to deploy the following open-source large language models (LLMs) locally, using the default half-precision format (FP16):

- Llama 3 8B-Instruct
- Llama 3 70B-instruct
- Mistral v0.2-Instruct
- Mixtral 8x7B v0.1-Instruct

LLM deployment and configuration

Each model was “prompted” to assess a report and determine whether it contained an abnormality. The simplest version of a prompt included a question (“is there an abnormality in this report”) and a report. For each report in a dataset, the prompt was updated and given to an LLM, which responded in natural language.

As the task was classification rather than text generation, we used model settings that lead to near-deterministic responses (see Supplementary Table 2 for specific parameters).

Prompt development

- Dataset: 2,179 labelled KCH reports (separate to the 545 reports held-out as a test set).
- Data split: 80:20 training-to-validation ratio, maintaining abnormal-to-normal label ratios.

We recursively, manually improved the prompt. After each improvement, the balanced accuracy was reviewed on both training and validation sets. Manual review of the responses was limited to the training set.

Prompt engineering techniques

- Chain-of-thought prompting [17]: we provided intermediate reasoning steps for the model to follow.
- Context inclusion: condensed label criteria for human labellers Supplementary Material 2) was included in the prompt.
- JSON output: we requested responses in JSON format, for consistency and for ease of parsing in Python.
- Few-shot prompting [18]: we included six example reports with ideal outputs (the maximum we could fit within the token limits).

LLM response processing

We used a Python script to automatically convert all LLM responses into a binary abnormality label. LLM responses from the hold-out test sets were not reviewed prior to reporting the final performance accuracies of each model.

Final prompt

You are a highly qualified expert trained to interpret radiology reports of MRI brains concisely. Your task is to briefly analyze the TEXT below and answer the question: based on the radiologist's interpretation of the images, does the report describe any intracranial or extracranial abnormalities anywhere in the brain or head? Answer "normal" if no abnormalities are described, and "abnormal" if at least one abnormality is described.
Base your decision of "normal" or "abnormal" on the description of the images written by the radiologist only. You must ignore the clinical context i.e. ignore the request made by the referring clinician, including the clinical details or clinical history. Do not answer with respect to the specific question to be answered. Do not let your answer be influenced by the clinical history/clinical details/question to be answered, and focus only on the radiologist's interpretation of the images.
Do not speculate whether other investigations or imaging would demonstrate that abnormality - if no abnormality is identified then this report is "normal". If findings are not seen in the scan, and no abnormal finding is described in the report, the report is "normal", even if all abnormalities cannot be fully excluded. The progression of findings should not influence your decision - an abnormality which is stable should still be labelled as "abnormal" e.g. a tumour decreasing in size from the previous study, or a post-surgical resection cavity. If there are findings that would not be found in the average healthy person (e.g. post-surgical resection cavity) label the text as "abnormal". If an incidental finding is not a normal variant, label the text as "abnormal". If no abnormality is described in the report, default to labelling the text as "normal", even when no other information is provided (e.g. research scans where they are not intended for clinical purposes, but no further description of the images has been given).

Specific criteria:
Small vessel disease (and the other names for it including small vessel ischaemia, small vessel ischaemic change, microangiopathy, SVD, microvascular disease) is considered "normal" only if it is described as mild. Mild-to-moderate, moderate, severe or confluent small vessel disease is considered "abnormal". If the type of small vessel disease is not described try to determine the severity from the description - moderate and worse small vessel disease requires confluence. White matter hyperintensities come under small vessel disease - if they are described as punctate, focal, or non-specific, they are considered "normal". If they are described as confluent, patchy, or extensive, they are considered "abnormal".

Any age-related atrophy (brain parenchymal volume loss) that is excessive for patient age is "abnormal", even if the excess is only slight. Patterns of volume loss that fit a specific subtype of dementia (e.g. frontotemporal, Alzheimer's) are considered "abnormal".
Most mass lesions including tumours are considered "abnormal". Most cysts are considered "abnormal" even if clinically insignificant or incidental (e.g. arachnoid cysts, Rathke cleft cysts, pineal cysts, choroid plexus cysts, retrocerebellar cysts are all "abnormal"). Perivascular (Virchow-Robin) spaces and Thornwalt cysts are the exception and are considered "normal".
All vascular abnormalities are considered "abnormal" e.g. aneurysms, arteriovenous malformations. Cavernomas, and developmental venous anomalies are also considered "abnormal" even if clinically insignificant or incidental. Normal anatomic variants are considered "normal", as are descriptions of sluggish flow, vascular fenestrations, flow-related signal abnormalities (unless they raise the suspicion of thrombus).
The following are categorised as "abnormal" encephalomalacia: gliosis, encephalomalacia, cavity, post-operative tissue changes/appearances, tumour debulking, partial resection, sequelae of infarct, sequelae of haemorrhage, cortical laminar necrosis, post-traumatic, or sequelae of any pathology or other injury. Post-surgical parenchymal changes should be considered "abnormal" even when the changes are expected or benign. Associated encephalomalacia-like findings which are considered "normal" unless there is a clear description of related parenchymal injury include craniotomy, burr-holes, posterior fossa decompression, and 3rd ventriculostomy.
Cerebellar ectopia and brain herniation are "abnormal".
Any extracranial abnormality should still be considered "abnormal", whatever the intracranial appearances. Mucosal thickening and retention cysts are considered "normal". Complete opacification/obstruction of the paranasal sinuses or a well-defined unambiguous polyp or mass is considered "abnormal". Hyperostosis is "normal".
The consequences of any previous pathology, e.g. signal abnormalities, are also considered "abnormal" (e.g. resection cavities, haemosiderin deposition), as they would not be present in the average healthy person.

You first REASON step by step:
1. Make a python list of findings in the report. Group multiple findings into one finding if they are all related to the same entity.
2. Make another python list of interpretations. For each finding, check if it matches the specific criteria provided for "abnormal" or "normal", and append the label to the interpretations list. If the finding is not listed in the criteria, then consider the overall description of the report and whether the average healthy person is likely to have that

finding, and label it as "abnormal" or "normal" accordingly and append this label to the interpretations list.
3. Make any reasoning for abnormal or normal decisions clear.
4. If any of the interpretations are "abnormal", then interpret the report as "abnormal".
5. If all the interpretations are "normal", or the INTERPRETATIONS list is empty, label the report as "normal".

You ALWAYS respond only in the following JSON format:

```
{
    "findings": [finding_1, finding_2...finding_n],
    "interpretation": [interpretation_1, interpretation_2...interpretation_n],
    "reason": "REASON",
    "label": "LABEL"
}
```

You only respond with one single JSON response.

Examples:
TEXT: [example report]
RESPONSE:

```
{
    "findings": ["Supravermian arachnoid cyst"],
    "interpretation": ["abnormal"],
    "reason": "Arachnoid cysts are considered abnormal in the criteria",
    "label": "abnormal"
}
```

TEXT: [example report]
RESPONSE:

```
{
    "findings": ["Post-operative changes secondary to meningioma resection", "residual T2 hyperintensity in the white matter"],
    "interpretation": ["abnormal", "normal"],
    "reason": "Post-operative changes are considered abnormal in the criteria. Unclear if residual T2 hyperintensity is abnormal",
    "label": "abnormal"
}
```

TEXT: [example report]
RESPONSE:

{
"findings": ["Post-operative changes secondary to tumour resection", "Some residual tumour component remaining"],
"interpretation": ["abnormal", "abnormal"],
"reason": "Post-operative changes and tumours are considered abnormal in the criteria",
"label": "abnormal"
}
TEXT: [example report]
RESPONSE:
{
"findings": ["Potentially an old ischaemic lesion in cerebellar hemispheres"],
"interpretation": ["abnormal"],
"reason": "Ischaemic lesions are abnormal and not found in the average healthy person",
"label": "abnormal"
}
TEXT: [example report]
RESPONSE:
{
"findings": [],
"interpretation": [],
"reason": "No abnormal findings described",
"label": "normal"
}
TEXT: [example report]
RESPONSE:
{
"findings": ["Mild small vessel ischaemic change"],
"interpretation": ["normal"],
"reason": "Mild small vessel disease is normal according to the criteria",
"label": "normal"
}
TEXT: [example report]
RESPONSE:
{
"findings": ["Resection cavity in right lateral temporal lobe", "minor small vessel ischaemic change"],
"interpretation": ["normal", "normal"],

"reason": "A resection cavity without parenchymal change, and mild small vessel disease are normal according to the criteria",
"label": "normal"
}

TEXT: [example report]

RESPONSE:

```
{
    "findings": ["Two subcutaneous lipomas in right frontal and left suboccipital region"],
    "interpretation": ["abnormal"],
    "reason": "Lipomas are mass lesions which are abnormal according to criteria",
    "label": "abnormal"
}
```

Your TEXT to analyze:

TEXT: [report]

Supplementary Table 2: Settings used for all large language models to perform binary abnormality classification of MRI brain reports

LLM parameter	Value
Number of responses	1
Temperature	0
Top p	1 (all tokens considered)
Top k	-1 (all tokens considered)
Maximum tokens	8192

LLM: large language model

7. Statistical analysis

7.1 Effect of masked language modelling dataset and BERT-based model choice on performance

Two-way analysis of variance (ANOVA) tests were performed on the KCH and GSTT hold-out test sets, where the dependent variable was balanced accuracy, and the independent variables were the choice of MLM dataset and the choice of BERT-based model. The choices of MLM dataset included no further MLM, MLM on unlabelled KCH reports and MLM on unlabelled KCH and GSTT reports. The effects of these factors and their interaction were assessed, and the results are shown in Supplementary Table 3.

Pairwise Tukey tests determined the overall effect of language modelling group on performance, shown in Supplementary Table 4.

One-way ANOVA tests were performed to investigate differences between BERT-based models, repeated for each language modelling dataset. The results are shown in Supplementary Table 5.

Ordinary least squares (OLS) regression was used as the underlying model to perform two-way ANOVA. Assumptions of normality and homogeneity of variances were met (Supplementary Tables 6 and 7 respectively).

Supplementary Table 3: Two-way ANOVA examining whether the choice of language modelling dataset, the choice of BERT-based model, or their interaction affects performance

	F-statistic, *p*-value	
	KCH hold-out test set	GSTT hold-out test set
Choice of MLM dataset	236.20, $p < 0.001$	169.67, $p < 0.001$
Choice of BERT-based model	6.58, $p < 0.001$	3.71, $p = 0.00133$
Interaction	5.10, $p < 0.001$	3.34, $p < 0.001$

MLM: Masked language modelling, BERT: Bidirectional Encoder Representations from Transformers, KCH: King's College Hospital, GSTT: Guys & St Thomas' Trust Hospital

Supplementary Table 4: Post-hoc Tukey tests, examining the effect of choice of language modelling dataset on performance (balanced accuracy)

Group 1 – Group 2	Mean difference (%) [95 CI]<br>*p*-value	
	KCH hold-out test set	GSTT hold-out test set
(KCH & GSTT MLM) – (No MLM)	+1.70 [1.43 – 1.97]<br>$p < 0.001$	+3.78 [3.15 – 4.41]<br>$p < 0.001$
(KCH MLM) – (No MLM)	+1.41 [1.14 – 1.68]<br>$p < 0.001$	+3.17 [2.54 – 3.79]<br>$p < 0.001$
(KCH & GSTT MLM) – (KCH MLM)	+0.29 [0.02 – 0.56]<br>$p = 0.0304$	+0.61 [-0.01 – 1.24]<br>$p = 0.0571$

MLM: Masked language modelling, KCH: King's College Hospital, GSTT: Guys & St Thomas' Trust Hospital

Supplementary Table 5: One-way ANOVA examining differences between BERT-based models across each language modelling variant

LM dataset	F-statistic, *p*-value	
	KCH hold-out test set	GSTT hold-out test set
KCH & GSTT MLM	1.81, $p = 0.121$	1.65, $p = 0.158$
KCH MLM	1.34, $p = 0.263$	2.11, $p = 0.0711$
No MLM	11.92, $p < 0.001$	5.26, $p < 0.001$

MLM: Masked language modelling, KCH: King's College Hospital, GSTT: Guys & St Thomas' Trust Hospital

Supplementary Table 6: Shapiro-Wilk tests for normality. All *p*-values were above the significance threshold of 0.05.

BERT-based model	Shapiro-Wilk statistic, *p*-value					
	KCH hold-out test set			GSTT hold-out test set		
	No MLM	KCH MLM	KCH & GSTT MLM	No MLM	KCH MLM	KCH & GSTT MLM
BERT (base)	0.824, *p = 0.*126	0.971, *p = 0.*880	0.987, *p = 0.*967	0.938, *p = 0.*653	0.948, *p = 0.722*	0.936, *p = 0.*635
RoBERTa (base)	0.892, *p = 0.*368	0.922, *p = 0.*546	0.874, *p = 0.*284	0.798, *p = 0.*078	0.929, *p = 0.*590	0.861, *p = 0.*231
BioBERT v1.1	0.885, *p = 0.*333	0.842, *p = 0.*171	0.867, *p = 0.*253	0.984, *p = 0.*957	0.894, *p = 0.*380	0.855, *p = 0.*211
SciBERT	0.886, *p = 0.*338	0.786, *p = 0.*062	0.953, *p = 0.*758	0.859, *p = 0.*226	0.837, *p = 0.*157	0.881, *p = 0.*316
BioClinicalBERT	0.972, *p = 0.*887	0.834, *p = 0.*148	0.939, *p = 0.*659	0.862, *p = 0.*235	0.783, *p = 0.*059	0.863, *p = 0.*238
PubMedBERT	0.975, *p = 0.*905	0.906, *p = 0.*444	0.866, *p = 0.*250	0.939, *p = 0.*655	0.913, *p = 0.*484	0.947, *p = 0.*712
UCSD RadBERT	0.971, *p = 0.*884	0.969, *p = 0.*871	0.963, *p = 0.*828	0.830, *p = 0.*140	0.963, *p = 0.*827	0.844, *p = 0.*178
Stanford RadBERT	0.934, *p = 0.*627	0.823, *p = 0.*123	0.947, *p = 0.*719	0.964, *p = 0.*838	0.907, *p = 0.*452	0.915, *p = 0.*501

MLM: Masked language modelling, BERT: Bidirectional Encoder Representations from Transformers, KCH: King's College Hospital, GSTT: Guys & St Thomas' Trust Hospital

Supplementary Table 7: Levene's test for homogeneity of variances. All *p*-values were above the significance threshold of 0.05.

F-statistic, *p*-value	
KCH hold-out test set	GSTT hold-out test set
0.54, *p = 0.*955	0.75, *p = 0.*779

KCH: King's College Hospital, GSTT: Guys & St Thomas' Trust Hospital

7.2 Ablation study: effect of language modelling dataset and number of reports on performance

Two-way analysis of variance (ANOVA) tests were performed on the KCH and GSTT hold-out test sets, where the dependent variable was balanced accuracy, and the independent variables were the choice of language modelling dataset and the number of reports. The effects of these factors and their interaction were assessed, and the results are shown in Supplementary Table 8.

Ordinary least squares (OLS) regression was used as the underlying model to perform two-way ANOVA. Assumptions of normality were met with a few exceptions (Supplementary Table 9). Assumptions of homogeneity of variances were met (Supplementary Table 10).

Supplementary Table 8: Two-way ANOVA examining whether the choice of language modelling dataset, the number of reports (log-transformed), or their interaction affects performance

	F-statistic, *p*-value	
	KCH hold-out test set	GSTT hold-out test set
Choice of MLM dataset	1.38, $p = 0.243$	2.17, $p = 0.142$
log(number of reports)	327.57, $p < 0.001$	176.53, $p < 0.001$
Interaction	0.138, $p = 0.711$	0.14, $p = 0.702$

MLM: Masked language modelling, BERT: Bidirectional Encoder Representations from Transformers, KCH: King’s College Hospital, GSTT: Guys & St Thomas’ Trust

Supplementary Table 9: Shapiro-Wilk tests for normality for the ablation study. *p*-values are marked with an asterisk (*) where below 0.05.

Percentage of MLM dataset used (%)	Shapiro-Wilk statistic, *p*-value			
	KCH hold-out test set		KCH hold-out test set	
	KCH MLM	KCH & GSTT MLM	KCH MLM	KCH & GSTT MLM
0	0.738, *p* = *0*.023*	0.738, *p* = *0*.023*	0.753, *p* = *0*.032*	0.753, *p* = *0*.032*
0.2	0.917, *p* = *0*.510	0.799, *p* = *0*.080	0.964, *p* = *0*.835	0.952, *p* = *0*.750
0.4	0.923, *p* = *0*.550	0.863, *p* = *0*.238	0.976, *p* = *0*.911	0.835, *p* = *0*.151
0.6	0.955, *p* = *0*.771	0.822, *p* = *0*.121	0.890, *p* = *0*.356	0.909, *p* = *0*.462
0.8	0.894, *p* = *0*.377	0.987, *p* = *0*.970	0.952, *p* = *0*.753	0.961, *p* = *0*.815
1	0.678, *p* = *0*.006	0.994, *p* = *0*.991	0.923, *p* = *0*.549	0.994, *p* = *0*.991
2	0.911, *p* = *0*.474	0.883, *p* = *0*.322	0.927, *p* = *0*.578	0.968, *p* = *0*.859
4	0.883, *p* = *0*.323	0.692, *p* = *0*.008*	0.901, *p* = *0*.417	0.893, *p* = *0*.370
6	0.953, *p* = *0*.759	0.907, *p* = *0*.448	0.882, *p* = *0*.317	0.960, *p* = *0*.808
8	0.970, *p* = *0*.874	0.748, *p* = *0*.028*	0.962, *p* = *0*.823	0.978, *p* = *0*.922
10	0.827, *p* = *0*.133	0.971, *p* = *0*.883	0.867, *p* = *0*.255	0.874, *p* = *0*.283
20	0.920, *p* = *0*.529	0.957, *p* = *0*.790	0.891, *p* = *0*.360	0.871, *p* = *0*.272
40	0.980, *p* = *0*.934	0.984, *p* = *0*.954	0.970, *p* = *0*.878	0.989, *p* = *0*.975
60	0.906, *p* = *0*.443	0.862, *p* = *0*.237	0.871, *p* = *0*.271	0.865, *p* = *0*.247
80	0.616, *p* = *0*.001*	0.705, *p* = *0*.011*	0.972, *p* = *0*.888	0.942, *p* = *0*.679
100	0.927, *p* = *0*.573	0.934, *p* = *0*.625	0.935, *p* = *0*.633	0.974, *p* = *0*.899

MLM: Masked language modelling, KCH: King’s College Hospital, GSTT: Guys & St Thomas’ Trust

Supplementary Table 10: Levene’s test for homogeneity of variances. All *p*-values were above the significance threshold of 0.05.

F-statistic, *p*-value	
KCH hold-out test set	GSTT hold-out test set
0.079, *p* = *0*.778	1.18, *p* = *0*.278

KCH: King’s College Hospital, GSTT: Guys & St Thomas’ Trust

8. Additional accuracy metrics

Supplementary Table 11: Extension of Table 3 - Performance of BERT-based models and LLMs for binary abnormality classification of brain MRI reports on KCH hold-out test set

		**Internal**				
**Name**	**Hospital Specific Domain Adaptation**	**F1 score**	**Precision**	**Recall**	**MCC**	**ROC-AUC**
BERT (base)	Both	96.47 ± 0.49	96.01 ± 0.53	96.95 ± 1.04	94.02 ± 0.82	99.62 ± 0.08
BERT (base)	KCH	96.37 ± 0.48	96.25 ± 0.59	96.50 ± 0.52	93.86 ± 0.81	99.49 ± 0.12
BERT (base)	Neither	93.37 ± 1.71	93.12 ± 1.18	93.63 ± 2.33	88.78 ± 2.84	98.69 ± 0.19
BioBERT v1.1	Both	96.43 ± 0.46	94.88 ± 0.58	98.03 ± 1.01	93.93 ± 0.79	99.64 ± 0.11
BioBERT v1.1	KCH	96.03 ± 0.24	95.48 ± 0.38	96.59 ± 0.83	93.27 ± 0.40	99.24 ± 0.22
BioBERT v1.1	Neither	94.91 ± 0.93	93.93 ± 2.13	95.96 ± 1.55	91.36 ± 1.59	99.17 ± 0.21
BioClinicalBERT	Both	96.99 ± 0.30	95.64 ± 0.52	98.39 ± 0.36	94.89 ± 0.50	99.74 ± 0.08
BioClinicalBERT	KCH	96.03 ± 0.54	94.65 ± 1.76	97.49 ± 0.97	93.26 ± 0.92	99.60 ± 0.09
BioClinicalBERT	Neither	94.48 ± 0.51	93.13 ± 1.13	95.87 ± 0.52	90.59 ± 0.89	99.16 ± 0.32
PubMedBERT	Both	96.31 ± 0.70	95.51 ± 0.86	97.13 ± 0.97	93.73 ± 1.19	99.57 ± 0.07
PubMedBERT	KCH	96.15 ± 0.64	94.78 ± 0.93	97.58 ± 1.08	93.46 ± 1.09	99.28 ± 0.24
PubMedBERT	Neither	94.46 ± 0.89	92.85 ± 1.71	96.14 ± 0.61	90.55 ± 1.54	99.05 ± 0.17

RoBERTa (base)	Both	96.54 ± 0.19	95.71 ± 1.23	97.40 ± 0.96	94.13 ± 0.34	99.30 ± 0.49
RoBERTa (base)	KCH	96.29 ± 0.49	94.95 ± 0.87	97.67 ± 0.91	93.69 ± 0.84	99.57 ± 0.06
RoBERTa (base)	Neither	94.01 ± 1.30	92.49 ± 1.94	95.61 ± 0.91	89.78 ± 2.23	99.02 ± 0.23
SciBERT	Both	96.42 ± 0.44	94.96 ± 0.77	97.94 ± 0.46	93.92 ± 0.76	99.67 ± 0.05
SciBERT	KCH	95.89 ± 0.48	95.48 ± 0.86	96.32 ± 0.72	93.04 ± 0.81	99.30 ± 0.11
SciBERT	Neither	94.59 ± 0.62	93.68 ± 1.22	95.52 ± 0.49	90.79 ± 1.07	99.06 ± 0.32
Stanford RadBERT	Both	96.59 ± 0.47	96.48 ± 1.45	96.77 ± 2.07	94.28 ± 0.78	99.71 ± 0.07
Stanford RadBERT	KCH	95.58 ± 0.36	95.21 ± 0.96	95.96 ± 1.10	92.51 ± 0.60	99.64 ± 0.08
Stanford RadBERT	Neither	94.62 ± 0.77	94.63 ± 0.95	94.62 ± 1.47	90.90 ± 1.29	99.12 ± 0.10
UCSD RadBERT	Both	96.48 ± 0.38	96.01 ± 0.60	96.95 ± 0.52	94.02 ± 0.65	99.46 ± 0.22
UCSD RadBERT	KCH	95.83 ± 0.79	94.94 ± 1.72	96.77 ± 0.44	92.92 ± 1.37	99.18 ± 0.65
UCSD RadBERT	Neither	95.52 ± 0.97	95.38 ± 1.39	95.70 ± 1.67	92.43 ± 1.64	99.28 ± 0.09

MLM: Masked language modelling, BERT: Bidirectional Encoder Representations from Transformers, RoBERTa: A Robustly Optimized BERT Pretraining Approach, UCSD: University of California San Diego, KCH: King's College Hospital, GSTT: Guys & St Thomas' Trust Hospital, LLM: Large language model. *LLM outputs were deterministic - multiple outputs would yield a standard deviation of zero.

Supplementary Table 12: Extension of Table 3 - Performance of BERT-based models and LLMs for binary abnormality classification of brain MRI reports on GSTT hold-out test set

Name	External Hospital Specific Domain Adaptation	F1 score	Precision	Recall	MCC	ROC-AUC
BERT (base)	Both	96.78 ± 0.80	96.03 ± 0.50	97.53 ± 1.19	92.89 ± 1.75	99.52 ± 0.24
BERT (base)	KCH	96.26 ± 0.92	94.19 ± 1.59	98.44 ± 0.66	91.69 ± 2.08	99.37 ± 0.26
BERT (base)	Neither	92.89 ± 1.58	89.61 ± 2.61	96.49 ± 2.20	84.04 ± 3.64	98.21 ± 0.45
BioBERT v1.1	Both	95.63 ± 0.71	93.33 ± 0.70	98.05 ± 0.92	90.27 ± 1.60	99.23 ± 0.15
BioBERT v1.1	KCH	95.62 ± 0.58	93.44 ± 0.79	97.92 ± 0.76	90.26 ± 1.31	99.28 ± 0.25
BioBERT v1.1	Neither	93.49 ± 0.54	89.56 ± 1.29	97.79 ± 0.88	85.38 ± 1.23	98.57 ± 0.14
BioClinicalBERT	Both	96.45 ± 0.50	96.13 ± 2.61	96.88 ± 1.81	92.28 ± 1.23	99.33 ± 0.18
BioClinicalBERT	KCH	93.94 ± 1.96	89.73 ± 3.99	98.70 ± 0.82	86.45 ± 4.43	99.16 ± 0.24
BioClinicalBERT	Neither	91.89 ± 0.83	87.33 ± 2.00	97.01 ± 0.97	81.67 ± 1.90	98.13 ± 0.45
PubMedBERT	Both	96.07 ± 0.75	94.09 ± 1.95	98.18 ± 1.12	91.29 ± 1.69	99.36 ± 0.18
PubMedBERT	KCH	94.66 ± 1.04	91.11 ± 2.65	98.57 ± 1.45	88.12 ± 2.35	99.13 ± 0.36
PubMedBERT	Neither	93.75 ± 0.59	90.67 ± 2.45	97.14 ± 1.91	86.06 ± 1.34	98.82 ± 0.17
RoBERTa (base)	Both	95.82 ± 1.98	93.59 ± 4.30	98.31 ± 0.88	90.72 ± 4.44	99.33 ± 0.19

RoBERTa (base)	KCH	95.81 ± 0.47	93.86 ± 2.17	97.92 ± 1.50	90.75 ± 1.09	99.15 ± 0.56
RoBERTa (base)	Neither	92.30 ± 1.25	88.38 ± 3.63	96.75 ± 1.69	82.68 ± 2.92	97.94 ± 0.68
SciBERT	Both	96.21 ± 0.53	95.32 ± 1.43	97.14 ± 1.20	91.64 ± 1.22	99.34 ± 0.27
SciBERT	KCH	95.37 ± 0.62	94.45 ± 1.69	96.36 ± 1.57	89.80 ± 1.39	99.32 ± 0.08
SciBERT	Neither	93.31 ± 0.86	90.08 ± 2.13	96.88 ± 2.41	85.07 ± 1.92	98.38 ± 0.39
Stanford RadBERT	Both	96.50 ± 0.44	94.65 ± 1.05	98.44 ± 0.88	92.24 ± 0.97	99.51 ± 0.14
Stanford RadBERT	KCH	95.72 ± 0.59	94.41 ± 2.18	97.14 ± 1.57	90.56 ± 1.34	99.25 ± 0.07
Stanford RadBERT	Neither	93.74 ± 1.29	91.68 ± 2.79	95.97 ± 1.39	86.03 ± 3.02	98.62 ± 0.43
UCSD RadBERT	Both	96.64 ± 0.55	96.18 ± 1.59	97.14 ± 1.20	92.63 ± 1.28	99.36 ± 0.37
UCSD RadBERT	KCH	95.74 ± 1.81	93.42 ± 3.83	98.31 ± 1.13	90.55 ± 4.07	99.24 ± 0.71
UCSD RadBERT	Neither	93.88 ± 0.95	91.48 ± 2.36	96.49 ± 1.77	86.36 ± 2.17	98.89 ± 0.27

MLM: Masked language modelling, BERT: Bidirectional Encoder Representations from Transformers, RoBERTa: A Robustly Optimized BERT Pretraining Approach, UCSD: University of California San Diego, KCH: King's College Hospital, GSTT: Guys & St Thomas' Trust Hospital, LLM: Large language model. *LLM outputs were deterministic - multiple outputs would yield a standard deviation of zero.

Supplementary Table 13: Extension to Table 4 - Performance of BERT-based models for multi-label classification of brain MRI reports.

**White matter inflammation**					
**Model**	**Hospital Specific Domain Adaptation**	**Precision**	**Recall**	**F1-score**	**ROC-AUC**
BioClinicalBERT	Both sites	96.06 ± 1.26	83.43 ± 1.14	89.30 ± 0.96	96.78 ± 0.89
BioClinicalBERT	None	84.96 ± 5.99	79.43 ± 3.79	81.82 ± 1.21	95.05 ± 1.14
PubMedBERT	Both sites	94.96 ± 4.25	83.43 ± 1.14	88.79 ± 2.30	98.26 ± 0.26
PubMedBERT	None	90.74 ± 4.03	82.29 ± 1.14	86.26 ± 1.96	96.59 ± 0.55
BERT (base)	Both sites	94.80 ± 1.53	82.86 ± 0.00	88.42 ± 0.66	97.47 ± 1.02
BERT (base)	None	78.80 ± 3.76	79.43 ± 2.14	79.01 ± 0.96	94.25 ± 0.35
BioBERT v1.1	Both sites	96.75 ± 2.89	82.86 ± 0.00	89.25 ± 1.23	97.07 ± 0.75
BioBERT v1.1	None	88.57 ± 3.92	78.29 ± 2.29	83.05 ± 2.03	95.48 ± 1.16
RoBERTa (base)	Both sites	99.33 ± 1.33	83.43 ± 1.14	90.68 ± 0.98	98.45 ± 0.33
RoBERTa (base)	None	78.98 ± 1.26	79.43 ± 1.14	79.20 ± 1.12	94.44 ± 0.64
SciBERT	Both sites	93.66 ± 3.84	82.29 ± 1.14	87.57 ± 1.83	98.03 ± 0.68
SciBERT	None	85.94 ± 2.86	80.00 ± 1.81	82.85 ± 2.12	96.68 ± 0.45
Stanford RadBERT	Both sites	96.00 ± 1.23	81.71 ± 2.29	88.26 ± 1.33	97.24 ± 0.58
Stanford RadBERT	None	80.43 ± 5.00	77.71 ± 3.33	78.88 ± 2.41	94.67 ± 0.59
UCSD RadBERT	Both sites	99.33 ± 1.33	82.86 ± 1.81	90.34 ± 1.21	97.94 ± 0.58
UCSD RadBERT	None	73.84 ± 1.25	80.57 ± 1.14	77.05 ± 0.94	93.36 ± 0.37
**Mass**					
**Model**	**Hospital Specific Domain Adaptation**	**Precision**	**Recall**	**F1-score**	**ROC-AUC**

BioClinicalBERT	Both sites	97.14 ± 2.34	73.33 ± 2.77	83.52 ± 1.75	96.79 ± 0.69
BioClinicalBERT	None	100.00 ± 0.00	68.89 ± 5.02	81.47 ± 3.55	95.15 ± 1.31
PubMedBERT	Both sites	96.27 ± 1.88	75.56 ± 3.78	84.60 ± 2.40	97.89 ± 0.36
PubMedBERT	None	92.65 ± 3.30	73.33 ± 4.91	81.75 ± 3.32	97.21 ± 1.21
BERT (base)	Both sites	96.32 ± 1.84	77.78 ± 2.34	86.05 ± 2.09	97.72 ± 1.05
BERT (base)	None	91.27 ± 5.09	43.70 ± 2.77	58.95 ± 1.87	93.14 ± 1.63
BioBERT v1.1	Both sites	97.26 ± 3.55	72.59 ± 3.78	83.01 ± 2.17	95.62 ± 0.41
BioBERT v1.1	None	96.99 ± 4.15	71.85 ± 5.02	82.49 ± 4.42	95.07 ± 0.15
RoBERTa (base)	Both sites	92.98 ± 5.34	70.37 ± 6.20	79.70 ± 2.66	95.79 ± 0.63
RoBERTa (base)	None	86.12 ± 2.59	68.15 ± 11.38	75.48 ± 7.59	92.59 ± 0.95
SciBERT	Both sites	98.05 ± 2.39	71.85 ± 1.81	82.90 ± 1.37	95.85 ± 0.91
SciBERT	None	81.96 ± 3.42	73.33 ± 2.77	77.35 ± 2.21	95.73 ± 1.01
Stanford RadBERT	Both sites	100.00 ± 0.00	70.37 ± 4.06	82.54 ± 2.86	96.40 ± 0.90
Stanford RadBERT	None	95.61 ± 2.28	65.19 ± 6.87	77.33 ± 5.41	97.22 ± 0.35
UCSD RadBERT	Both sites	92.66 ± 3.30	73.33 ± 2.77	81.81 ± 2.02	96.21 ± 0.85
UCSD RadBERT	None	100.00 ± 0.00	67.41 ± 4.32	80.45 ± 3.06	94.88 ± 0.89
**Atrophy**					
**Model**	**Hospital Specific Domain Adaptation**	**Precision**	**Recall**	**F1-score**	**ROC-AUC**
BioClinicalBERT	Both sites	97.07 ± 0.98	88.44 ± 2.59	92.54 ± 1.63	98.43 ± 0.45
BioClinicalBERT	None	94.12 ± 2.20	83.11 ± 7.38	88.03 ± 4.06	97.30 ± 0.34
PubMedBERT	Both sites	97.68 ± 2.06	91.11 ± 1.41	94.26 ± 0.69	99.37 ± 0.14
PubMedBERT	None	94.66 ± 2.10	93.33 ± 1.41	93.97 ± 0.85	98.45 ± 0.86

BERT (base)	Both sites	97.67 ± 1.44	91.56 ± 1.66	94.49 ± 0.49	99.51 ± 0.11
BERT (base)	None	93.12 ± 1.25	89.78 ± 2.27	91.39 ± 1.00	97.45 ± 0.77
BioBERT v1.1	Both sites	99.04 ± 1.18	90.67 ± 1.66	94.66 ± 1.20	99.61 ± 0.23
BioBERT v1.1	None	91.75 ± 2.29	87.56 ± 5.37	89.47 ± 2.52	98.66 ± 0.46
RoBERTa (base)	Both sites	97.12 ± 1.78	88.89 ± 1.41	92.81 ± 1.13	99.22 ± 0.18
RoBERTa (base)	None	87.79 ± 2.14	89.33 ± 2.59	88.54 ± 2.06	97.12 ± 0.66
SciBERT	Both sites	98.05 ± 0.98	88.44 ± 0.89	92.99 ± 0.07	98.85 ± 0.33
SciBERT	None	90.74 ± 0.16	87.11 ± 1.66	88.88 ± 0.95	97.00 ± 0.85
Stanford RadBERT	Both sites	98.06 ± 0.97	89.78 ± 1.09	93.73 ± 0.94	99.38 ± 0.21
Stanford RadBERT	None	94.38 ± 1.70	80.89 ± 4.12	87.03 ± 2.00	96.44 ± 0.72
UCSD RadBERT	Both sites	96.71 ± 1.12	91.11 ± 3.14	93.80 ± 1.79	99.19 ± 0.17
UCSD RadBERT	None	94.52 ± 0.98	91.56 ± 2.95	92.98 ± 1.43	98.44 ± 0.26
**Vascular**					
**Model**	**Hospital Specific Domain Adaptation**	**Precision**	**Recall**	**F1-score**	**ROC-AUC**
BioClinicalBERT	Both sites	80.29 ± 10.23	53.33 ± 12.96	62.16 ± 7.94	97.40 ± 0.21
BioClinicalBERT	None	55.33 ± 15.72	42.22 ± 17.78	46.72 ± 15.49	90.85 ± 4.25
PubMedBERT	Both sites	82.29 ± 9.46	46.67 ± 4.44	59.09 ± 2.41	98.52 ± 0.27
PubMedBERT	None	64.62 ± 8.87	35.56 ± 10.89	44.97 ± 9.73	92.03 ± 1.62
BERT (base)	Both sites	91.43 ± 11.43	57.78 ± 8.31	70.09 ± 7.14	98.72 ± 0.56
BERT (base)	None	51.33 ± 8.59	17.78 ± 5.44	25.81 ± 6.41	80.76 ± 1.43
BioBERT v1.1	Both sites	86.48 ± 7.00	55.56 ± 7.03	67.38 ± 6.00	98.40 ± 0.60
BioBERT v1.1	None	82.00 ± 1.63	51.11 ± 5.44	62.86 ± 4.67	93.44 ± 1.28

RoBERTa (base)	Both sites	68.57 ± 2.33	53.33 ± 8.31	59.67 ± 5.39	96.82 ± 0.68
RoBERTa (base)	None	48.77 ± 11.00	44.44 ± 12.17	46.11 ± 10.77	86.04 ± 4.19
SciBERT	Both sites	97.14 ± 5.71	57.78 ± 8.31	71.88 ± 6.05	98.40 ± 0.52
SciBERT	None	88.33 ± 14.53	26.67 ± 8.89	39.90 ± 11.68	93.49 ± 1.55
Stanford RadBERT	Both sites	84.67 ± 14.85	44.44 ± 9.94	57.62 ± 10.48	97.52 ± 0.57
Stanford RadBERT	None	45.33 ± 10.46	26.67 ± 5.44	33.39 ± 6.79	89.22 ± 1.49
UCSD RadBERT	Both sites	67.71 ± 9.27	46.67 ± 8.31	55.07 ± 8.57	97.19 ± 0.45
UCSD RadBERT	None	57.90 ± 8.18	44.44 ± 14.05	49.53 ± 12.11	94.34 ± 2.36
**Stroke**					
**Model**	**Hospital Specific Domain Adaptation**	**Precision**	**Recall**	**F1-score**	**ROC-AUC**
BioClinicalBERT	Both sites	83.33 ± 0.00	100.00 ± 0.00	90.91 ± 0.00	99.85 ± 0.10
BioClinicalBERT	None	72.00 ± 6.53	80.00 ± 0.00	75.64 ± 3.56	98.00 ± 1.16
PubMedBERT	Both sites	83.33 ± 0.00	100.00 ± 0.00	90.91 ± 0.00	99.64 ± 0.15
PubMedBERT	None	70.95 ± 6.46	88.00 ± 9.80	78.48 ± 7.45	99.58 ± 0.32
BERT (base)	Both sites	76.19 ± 5.83	100.00 ± 0.00	86.36 ± 3.71	99.85 ± 0.17
BERT (base)	None	73.00 ± 8.59	76.00 ± 14.97	74.06 ± 10.71	99.12 ± 0.29
BioBERT v1.1	Both sites	83.33 ± 0.00	100.00 ± 0.00	90.91 ± 0.00	99.91 ± 0.12
BioBERT v1.1	None	68.85 ± 9.38	100.00 ± 0.00	81.19 ± 6.59	99.82 ± 0.11
RoBERTa (base)	Both sites	83.33 ± 0.00	100.00 ± 0.00	90.91 ± 0.00	99.82 ± 0.11
RoBERTa (base)	None	80.33 ± 10.97	64.00 ± 14.97	70.33 ± 11.27	99.42 ± 0.11
SciBERT	Both sites	83.33 ± 0.00	100.00 ± 0.00	90.91 ± 0.00	99.76 ± 0.12
SciBERT	None	76.33 ± 5.21	76.00 ± 8.00	75.88 ± 5.40	98.39 ± 1.56

Stanford RadBERT	Both sites	82.67 ± 1.33	96.00 ± 8.00	88.73 ± 4.36	99.76 ± 0.07
Stanford RadBERT	None	80.00 ± 6.67	96.00 ± 8.00	87.27 ± 7.27	99.79 ± 0.21
UCSD RadBERT	Both sites	83.33 ± 0.00	100.00 ± 0.00	90.91 ± 0.00	99.61 ± 0.12
UCSD RadBERT	None	72.00 ± 6.53	80.00 ± 0.00	75.64 ± 3.56	99.39 ± 0.27
**Small vessel disease**					
**Model**	**Hospital Specific Domain Adaptation**	**Precision**	**Recall**	**F1-score**	**ROC-AUC**
BioClinicalBERT	Both sites	93.19 ± 2.22	86.36 ± 2.87	89.61 ± 1.94	96.51 ± 0.94
BioClinicalBERT	None	69.79 ± 14.59	48.18 ± 13.67	56.40 ± 12.77	90.59 ± 2.50
PubMedBERT	Both sites	95.29 ± 4.78	86.36 ± 4.07	90.48 ± 2.98	98.93 ± 0.74
PubMedBERT	None	81.19 ± 5.19	76.36 ± 4.45	78.51 ± 2.94	93.62 ± 1.01
BERT (base)	Both sites	95.09 ± 3.02	86.36 ± 2.87	90.47 ± 2.14	98.41 ± 0.31
BERT (base)	None	73.80 ± 9.13	62.73 ± 6.68	67.68 ± 7.10	91.73 ± 2.63
BioBERT v1.1	Both sites	95.09 ± 3.02	84.55 ± 4.64	89.37 ± 2.14	97.71 ± 1.24
BioBERT v1.1	None	88.45 ± 3.56	77.27 ± 11.85	82.03 ± 8.12	95.68 ± 1.86
RoBERTa (base)	Both sites	90.59 ± 3.80	85.45 ± 1.82	87.88 ± 1.52	96.24 ± 0.94
RoBERTa (base)	None	74.21 ± 8.03	61.82 ± 12.40	65.96 ± 6.18	90.74 ± 1.21
SciBERT	Both sites	97.99 ± 2.46	88.18 ± 3.64	92.80 ± 2.72	97.99 ± 1.24
SciBERT	None	82.71 ± 8.81	62.73 ± 7.27	71.07 ± 6.70	95.23 ± 1.00
Stanford RadBERT	Both sites	94.94 ± 3.17	84.55 ± 3.64	89.41 ± 2.92	98.59 ± 0.44
Stanford RadBERT	None	71.31 ± 3.79	50.00 ± 4.98	58.72 ± 4.58	90.38 ± 2.38
UCSD RadBERT	Both sites	90.39 ± 4.13	84.55 ± 6.17	87.25 ± 4.29	95.42 ± 0.97
UCSD RadBERT	None	77.27 ± 3.03	72.73 ± 7.04	74.60 ± 3.15	95.40 ± 0.83

Encephalomalacia					
**Model**	**Hospital Specific Domain Adaptation**	**Precision**	**Recall**	**F1-score**	**ROC-AUC**
BioClinicalBERT	Both sites	91.04 ± 4.36	90.00 ± 1.82	90.46 ± 2.45	98.86 ± 0.34
BioClinicalBERT	None	79.66 ± 6.59	80.91 ± 6.03	79.85 ± 2.20	95.90 ± 1.22
PubMedBERT	Both sites	97.19 ± 2.30	91.82 ± 1.82	94.40 ± 1.12	97.85 ± 0.69
PubMedBERT	None	82.25 ± 7.11	92.73 ± 2.23	86.96 ± 3.43	99.04 ± 0.24
BERT (base)	Both sites	87.13 ± 4.17	90.00 ± 3.40	88.41 ± 1.53	98.76 ± 0.14
BERT (base)	None	70.81 ± 7.55	71.82 ± 4.45	70.99 ± 3.94	94.09 ± 0.99
BioBERT v1.1	Both sites	89.76 ± 3.34	87.27 ± 4.45	88.45 ± 3.38	98.60 ± 0.54
BioBERT v1.1	None	78.49 ± 6.72	89.09 ± 3.64	83.19 ± 3.26	97.70 ± 0.35
RoBERTa (base)	Both sites	84.39 ± 5.28	90.00 ± 3.40	86.92 ± 2.24	98.74 ± 0.45
RoBERTa (base)	None	73.40 ± 5.83	79.09 ± 15.37	75.30 ± 9.36	95.75 ± 2.04
SciBERT	Both sites	90.51 ± 4.43	83.64 ± 3.64	86.80 ± 2.28	98.26 ± 0.49
SciBERT	None	80.32 ± 7.36	80.00 ± 5.45	80.08 ± 6.03	97.29 ± 0.75
Stanford RadBERT	Both sites	91.85 ± 4.54	88.18 ± 2.23	89.86 ± 1.46	98.93 ± 0.73
Stanford RadBERT	None	74.16 ± 5.28	71.82 ± 7.27	72.72 ± 4.92	96.32 ± 0.44
UCSD RadBERT	Both sites	88.22 ± 4.33	92.73 ± 2.23	90.31 ± 1.52	98.96 ± 0.36
UCSD RadBERT	None	82.99 ± 2.62	92.73 ± 2.23	87.56 ± 2.04	97.67 ± 0.99

MLM: Masked language modelling, BERT: Bidirectional Encoder Representations from Transformers, RoBERTa: A Robustly Optimized BERT Pretraining Approach, UCSD: University of California San Diego

9. Example reports and classifications

Here we demonstrate some failure cases between the UCSD-RadBERT model. UCSD-RadBERT was chosen as it was already pretrained with MLM on 4 million reports from the US, so we can compare the effect of additional hospital-specific domain adaptation. We compare models finetuned for abnormality classification on fold 1 of the internal hold-out test set; one variant with hospital-specific domain adaptation and one without.

Reports where UCSD-RadBERT with hospital-specific domain adaptation (HSDA) incorrect and base UCSD-RadBERT correct (n=6):

Report: Clinical History : Clinical Details: TIA protocol. sudden onset R sided Face / arm / leg numbness lasting some hours. ABCD score 3. RFs HTN. Patient will be on David Marsden ward at 7.30 Specific question to be answered: ? restricted diffusion
MRI Head : There is no significant white matter ischaemic load. No acutely restricted lesions are demonstrated. There is a tiny focus of low signal within the right thalamus on the gradient echo-T2* sequences. The parenchymal appearances are otherwise normal. The intracranial flow related signal voids are within normal limits.
Label: 1
Prediction UCSD-RadBERT with HSDA: 0 (FN)
Prediction UCSD-RadBERT no HSDA: 1 (TP)

Report: Clinical History : Traumatic head injury in the past. Now unstable mental state: Manic, reports auditory hallucinations in the past. Any change to his brain injury, could it explain his symptoms?
MRI Head : Axial T2, flair, T2 star, volume T1 with reformats. There no previous studies comparison. There are normal intracranial appearances. There is no evidence of previous intracranial haemorrhage or focal and carefully malacic change. There is no focal mass or abnormal white matter signal. There is no specific neurodegenerative pattern. There is moderate mucosal thickening of the left maxillary sinus with a small fluid level. Adenoidal prominence is noted which is slightly unusual for a patient of this age. Clinical correlation is recommended. Conclusion: There are normal intracranial appearances. A clinical review of

the posterior nasal spaces adenoidal tissue is recommended. U coded as requested by referrer.
Label: 1
Prediction UCSD-RadBERT with HSDA: 0 (FN)
Prediction UCSD-RadBERT no HSDA: 1 (TP)

Report: Clinical Details: pre DBS. Parkinson's disease with right flapping tremor.DBS May need SXR before Specific question to be answered: arophy, or basal ganglia abnormality
MRI Head : Axial dual echo, axial T2 gradient echo, diffusion and coronal T1 volume sequences obtained. There are moderate generalised involutional changes in keeping with age. Minor patchy white matter T2 hyperintensity is seen within the frontal and parietal lobes and is most probably related to minor small vessel disease. There are prominent perivascular spaces within the cerebral hemispheres and basal ganglia bilaterally. The basal ganglia volume is preserved. There is no cortical infarction. There is no evidence of significant basal ganglia minimalisation. Thalami brain stem and cerebellum are preserved.
CONCLUSION: Generalised cerebral involutional change and minor small vessel disease. Prominent perivascular spaces in the basal ganglia, a normal variant. No major basal ganglia or brain stem abnormality.
Label: 1
Prediction UCSD-RadBERT with HSDA: 0 (FN)
Prediction UCSD-RadBERT no HSDA: 1 (TP)

Report: Clinical Details: severe headache, blurring of vision, difficulty with swallow and problems with balance Specific question to be answered: headaches are suggestive of migraine, but is there any evidence of brainstem involvement
MRI Head : Ax T2W, Cor FLAIR, Sag T1W, DWI and MRV were obtained. The intracranial appearances are normal, specifically the posterior fossa appears normal (incidental note is made of a prominent VR space in the left lentiform nucleus.) The dural venous sinuses appear normal. Extensive mucosal thickening is shown involving the maxillary antrum, ethmoid aircells, frontal sinus and sphenoid sinus on the right. Fluid is also shown within the mastoid air cells on the right.
Label: 1
Prediction UCSD-RadBERT with HSDA: 0 (FN)
Prediction UCSD-RadBERT no HSDA: 1 (TP)

Report: MRI Head : T2 Axial, Coronal FLAIR, T1 Sagittal images of the Brain with Diffusion imaging. There is 3mm of cerebellar tonsillar descent without evidence of compaction. The

ventricles are of normal size. There is no cerebral signal abnormality. The features are unchanged relative to the MRI of 10/05. In Conclusion: There are normal intracranial MRI appearances.
Label: 0
Prediction UCSD-RadBERT with HSDA: 1 (FP)
Prediction UCSD-RadBERT no HSDA: 0 (TN)

Report: MRI Functional imaging : MR Head IOP Research scan. These contain limited sequences. Not to be used for clinical purposes. Mild cerebellar tonsillar ectopia. Referral to GP not required.
Label: 0
Prediction UCSD-RadBERT with HSDA: 1 (FP)
Prediction UCSD-RadBERT no HSDA: 0 (TN)

Reports where UCSD-RadBERT with hospital-specific domain adaptation correct and base UCSD-RadBERT incorrect (n=12):

Report: Clinical Details: Discharged from HASU 10/12 for Right MCA infarct thrombolysed on 9/12/18- NIHSS = 7. Discharge NIHSS 10/12 = 1 (left arm)\.br\Presented with slurred speech, facial palsy and left sided arm weakness was worse NIHSS = 5 on 11/12 CTH nil new findings Specific question to be answered: ? stroke
MRI brain The structural and diffusion sequences would suggest a single age of subacute right hemispheric MCA embolic injury.
Label: 1
Prediction UCSD-RadBERT with HSDA: 1 (TP)
Prediction UCSD-RadBERT no HSDA: 0 (FN)

Report: Clinical Details: worsening headache peri and post partum. gave birth 6/7 ago
Specific question to be answered: ? venous sinus thrombosis - needs MRV please
MRI Brain Axial T2, DWI, coronal flair, sagittal T1, non-contrast enhanced MRV. Report: The intracranial appearances are normal. There is no evidence of parenchymal signal abnormality, and no ancillary features to suggest a venous sinus thrombosis. The left transverse sinus is hypoplastic, and therefore not well visualised on the MRV. The craniocervical junction is normal. Note is made of moderate mucosal thickening and retention of secretions in the sphenoid sinuses bilaterally with some lateral pneumatised aeration of the base of sphenoid. The ethmoid sinuses and maxillary sinuses are clear.

Conclusion: No evidence of venous sinus thrombosis. Normal intracranial appearances. Sphenoid sinus inflammation. If there is a concern regarding subarachnoid haemorrhage then an LP should be performed.
Label: 0
Prediction UCSD-RadBERT with HSDA: 0 (TN)
Prediction UCSD-RadBERT no HSDA: 1 (FP)

Report: Clinical History : Clinical Details: c/o neck pain. spastic tetraparesis Specific question to be answered: ?cord lesion
MRI Head : Sequences obtained: Coronal FLAIR images only. There is a small solitary focus of high signal seen in the left posterior corona radiata. We do not have further sequences to characterise this further and this is unlikely to be of clinical significance..
Label: 0
Prediction UCSD-RadBERT with HSDA: 0 (TN)
Prediction UCSD-RadBERT no HSDA: 1 (FP)

Report: MRI HEAD Comparison was made with the previous scan of 9/4/2008. There has been no change in the scan appearances or the degree of dilatation of the ventricular system. Flow voids are again noted through the aqueduct and exit foramina of the fourth ventricle.
Label: 1
Prediction UCSD-RadBERT with HSDA: 1 (TP)
Prediction UCSD-RadBERT no HSDA: 0 (FN)

Report: Clinical Details: 33 yr old - run over while on bike and then punched by driver (perforated ear drum) and LOC. Continued severe headaches esp morning, unable to concentrate and work. Also temp control affected - shivers/ sweats Specific question to be answered: ? any structural abn from head injury. Also any abn around hypothalamus
MRI Head : Axial T2w/T2*, Coronal FLAIR, Sagittal T1w, DWI. There are 7-8 scattered superficial and deep white matter T2 hyperintense foci within the cerebral hemispheres, which are non-specific in aetiology. A tiny rounded focus of T2 low signal is seen in the anteroinferior left temporal region, in a sulcal location. This may represent a small focus of calcification or haemosiderin. Otherwise normal intracranial appearances.
Label: 1
Prediction UCSD-RadBERT with HSDA: 1 (TP)
Prediction UCSD-RadBERT no HSDA: 0 (FN)

Report: Clinical History : epilepsy of upper limbs and whole body.
MRI Head : Normal intracranial appearances. Note is made of generalised cerebral and cerebellar volume loss in excess of the patient's age.
Label: 1
Prediction UCSD-RadBERT with HSDA: 1 (TP)
Prediction UCSD-RadBERT no HSDA: 0 (FN)

Report: MRI BRAIN Axial T2, axial FLAIR, sagittal T2, coronal T1 pre and post contrast, axial MTP post contrast. The multiple white matter lesions remain typical for, and fulfill imaging criteria for multiple sclerosis. I can find no new lesion or new enhancement in comparison to 31/07/2008.
Label: 1
Prediction UCSD-RadBERT with HSDA: 1 (TP)
Prediction UCSD-RadBERT no HSDA: 0 (FN)

Report: Clinical Details: HCC for palliative care, recent h/o seizures- seen at PRUH- CT head showed ? old bleed Specific question to be answered: to look for any brain mets
MRI Head : No space occupying lesion seen. There are thin bilateral subdural collections (up to4 m in depth on the left).No restricted diffusion demonstrated. Minor patchy deep white matter low attenuation in keeping with mild small vessel disease. Normal appearances of the ventricles and sulci.Gadolinium would be required to fully evaluation for intracranial metastasis.Normal calvarial and skull base appearances. Impression: No evidence of brain metastases.
Label: 1
Prediction UCSD-RadBERT with HSDA: 1 (TP)
Prediction UCSD-RadBERT no HSDA: 0 (FN)

Report: Clinical Details: 34 male with temporal lobe epilepsy. Suspicion of left hippocampal sclerosis on previous MRIs from Cardif. Patient having video telemetry but needs MRI once disconnected towards end of this week please. Specific question to be answered: ?Hippocampal Sclerosis
MRI Head : Ax T2W, Cor T1 Volume, Hi res Cor T2W, Cor FLAIR, DWI sequences were obtained. The left hippocampus is small and of T2 high signal, indicating hippocampal sclerosis. The intracranial appearances are otherwise normal.
Label: 1
Prediction UCSD-RadBERT with HSDA: 1 (TP)
Prediction UCSD-RadBERT no HSDA: 0 (FN)

Report: Clinical History :
MRI Head : Epilepsy protocol scan acquired using T2 and DWI axials and T2, FLAIR and volume T1 coronals. There is a very small focus of T2 and FLAIR high signal with corresponding low T1 signal within the right frontal subcortical white matter in the pre central gyrus. This lesion closely abuts the deep surface of the cortex but the overlying cortex is largely preserved. The intracranial appearances are otherwise preserved. CONCLUSION: Small non specific subcortical white matter focus in the right frontal lobe. No other intracranial abnormality of note.
Label: 0
Prediction UCSD-RadBERT with HSDA: 0 (TN)
Prediction UCSD-RadBERT no HSDA: 1 (FP)

Report: Clinical History : Clinical Details: Left sided weakness.Post-thrombolysis CT did not show a stroke or haemorrhage. Now complaints of pain in L leg with left sided weakness.OE:Pronator drift L arm .MRI today in view of persisiting symptoms and 2 normal scans Specific question to be answered: MRI in view of persisiting symptoms and 2 normal scans.to r/o any infarct/bleed/. Needs transport booked?: No
MRI Head : Axial T2W and T2* GRE and DWI obtained. There is restricted cortical diffusion in the right paracentral lobule, most likely in distal ACA territory. There is no evidence of any other ischaemic or haemorrhagic event or other significant intracranial abnormality.
Label: 1
Prediction UCSD-RadBERT with HSDA: 1 (TP)
Prediction UCSD-RadBERT no HSDA: 0 (FN)

Report: MRI Head : T2/FLAIR axial, volumetric T1 MP RAGE sagittal images. T2 * axial images. There are a number (at least 35) of hemispheric white matter T2 hyperintensities. These are principally within the frontal lobes and within the subcortical white matter. The ventricles and CSF spaces about the brain are of normal configuration. There is a mild degree of plagiocephaly. There is no evidence of haemorrhagic degradation products on the T2 * imaging. There is no frontal lobe cortical abnormality. CONCLUSION There are a number of hemispheric white matter T2 hyperintense lesions which are non-specific, although a vascular aetiology is felt more likely than an inflammatory/demyelinating or post traumatic origin.
Label: 1
Prediction UCSD-RadBERT with HSDA: 1 (TP)
Prediction UCSD-RadBERT no HSDA: 0 (FN)

Supplementary material references

1. Bandy J, Vincent N Addressing "Documentation Debt" in Machine Learning: A Retrospective Datasheet for BookCorpus

2. Devlin J, Chang M-W, Lee K, Toutanova K (2019) BERT: Pre-training of Deep Bidirectional Transformers for Language Understanding. In: Burstein J, Doran C, Solorio T (eds) Proceedings of the 2019 Conference of the North American Chapter of the Association for Computational Linguistics: Human Language Technologies, Volume 1 (Long and Short Papers). Association for Computational Linguistics, Minneapolis, Minnesota, pp 4171–4186

3. Wood DA, Kafiabadi S, Al Busaidi A, et al (2022) Deep learning to automate the labelling of head MRI datasets for computer vision applications. Eur Radiol 32:725–736. https://doi.org/10.1007/s00330-021-08132-0

4. Fazekas F, Chawluk JB, Alavi A, et al (1987) MR signal abnormalities at 1.5 T in Alzheimer's dementia and normal aging. AJR Am J Roentgenol 149:351–356. https://doi.org/10.2214/ajr.149.2.351

5. Liu Y, Ott M, Goyal N, et al (2019) RoBERTa: A Robustly Optimized BERT Pretraining Approach

6. (2024) google-research/bert

7. Akiba T, Sano S, Yanase T, et al (2019) Optuna: A Next-generation Hyperparameter Optimization Framework. In: Proceedings of the 25th ACM SIGKDD International Conference on Knowledge Discovery & Data Mining. Association for Computing Machinery, New York, NY, USA, pp 2623–2631

8. Loshchilov I, Hutter F (2019) Decoupled Weight Decay Regularization

9. Devlin J, Chang M-W, Lee K, Toutanova K (2019) BERT: Pre-training of Deep Bidirectional Transformers for Language Understanding

10. Lee J, Yoon W, Kim S, et al (2020) BioBERT: a pre-trained biomedical language representation model for biomedical text mining. Bioinformatics 36:1234–1240. https://doi.org/10.1093/bioinformatics/btz682

11. Beltagy I, Lo K, Cohan A (2019) SciBERT: A Pretrained Language Model for Scientific Text

12. Alsentzer E, Murphy JR, Boag W, et al (2019) Publicly Available Clinical BERT Embeddings

13. Gu Y, Tinn R, Cheng H, et al (2021) Domain-Specific Language Model Pretraining for Biomedical Natural Language Processing. ACM Trans Comput Healthcare 3:2:1-2:23. https://doi.org/10.1145/3458754

14. Yan A, McAuley J, Lu X, et al (2022) RadBERT: Adapting Transformer-based Language Models to Radiology. Radiology: Artificial Intelligence 4:e210258. https://doi.org/10.1148/ryai.210258

15. Chambon P, Cook TS, Langlotz CP (2023) Improved Fine-Tuning of In-Domain Transformer Model for Inferring COVID-19 Presence in Multi-Institutional Radiology Reports. J Digit Imaging 36:164–177. https://doi.org/10.1007/s10278-022-00714-8

16. Kwon W, Li Z, Zhuang S, et al (2023) Efficient Memory Management for Large Language Model Serving with PagedAttention

17. Wei J, Wang X, Schuurmans D, et al (2023) Chain-of-Thought Prompting Elicits Reasoning in Large Language Models

18. Brown T, Mann B, Ryder N, et al (2020) Language Models are Few-Shot Learners. In: Advances in Neural Information Processing Systems. Curran Associates, Inc., pp 1877–1901
